# Clinical Features and *PLCZ1* Gene Variants in Two Cases of Male Infertility: A Case Series and Literature Review

**DOI:** 10.1002/mgg3.70250

**Published:** 2026-06-15

**Authors:** Jinwei Yang, Bo Yan, Zhizhuo Wei, Zijing Niu, Tongguang Wang, Yan Wei, Fangzhu Wang, Wei Jiang, Lin Zhang, Yue Da, Huanjing Wang, Yali Ni, Zhiqiang Wang

**Affiliations:** ^1^ Reproductive Medical Center Gansu Provincial Maternity and Child‐Care Hospital Lanzhou Gansu China; ^2^ Key Laboratory of Maternal‐Fetal Medicine and Reproductive Protection of Gansu Province Lanzhou Gansu Province China; ^3^ Gansu University of Chinese Medicine Lanzhou Gansu China; ^4^ The Second Hospital of Lanzhou University Lanzhou Gansu China; ^5^ Department of Physiology and Pathophysiology, School of Basic Medical Sciences Xi'an Jiaotong University Xi'an Shaanxi Province China

**Keywords:** assisted oocyte activation (AOA), fertilization failure, male infertility, *PLCZ1*, whole‐exome sequencing (WES)

## Abstract

**Background:**

Variations in the *PLCZ1* gene can lead to male infertility (MIM #617214), an autosomal recessive disorder characterized by failure or abnormal fertilization of oocytes after intracytoplasmic sperm injection. This condition can be addressed using assisted oocyte activation. Although various mutation types have been reported, our study identified two novel pathogenic variants.

**Methods:**

Whole‐exome sequencing (WES) was performed to screen for pathogenic variants. Candidate variants were validated using Sanger sequencing and pedigree analysis. Pathogenicity was assessed with bioinformatics tools and the ACMG guidelines. A literature review on *PLCZ1* mutations was conducted using PubMed, CNKI, and Wanfang databases.

**Results:**

Our results showed that both patients carried homozygous frameshift pathogenic variants in *PLCZ1*: c.138_139delCA (p. D46Efs*2) (Family 1, II‐2) and c.1087del (p. S363Afs*64) (Family 2, II‐2). The parents of family 1 were both heterozygous carriers, which was consistent with the autosomal recessive inheritance pattern. For family 2, the parents were deceased, so validation could not be performed. The clinical intervention results of the two patients showed that almost all fertilizations failed in the first cycle, and after assisted oocyte activation in the second cycle, the normal fertilization rates increased to 72.7% and 80.0% respectively, and both couples eventually gave birth to healthy offspring. In the retrospective analysis of the literature, 24 studies and 77 patients with 46 different genetic variants were included. Missense mutations were the most common 63.1% (65/103), and exon 6 was the mutation hotspot 31.0% (32/103).

**Conclusion:**

This study reported for the first time that homozygous frameshift mutations in the *PLCZ1* gene led to fertilization failure. These newly discovered mutations expanded the range of pathogenic variants in the *PLCZ1* gene, confirming that ICSI combined with assisted oocyte activation can effectively overcome such male factor infertility, providing a theoretical basis for genetic counseling and reproductive intervention for such patients.

## Graphical abstract

Our study expands the variant spectrum of PLCZ1, enriches the clinical characteristics of PLCZ1, and provides a solid base for the genetic counseling, diagnosis, and treatment of this condition.

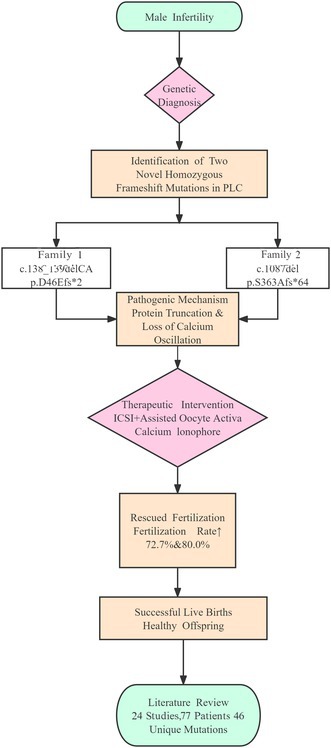

## 1 Introduction

Infertility is a common and serious condition affecting human reproductive health, and its incidence is increasing year by year. It affects approximately 15% of couples of reproductive age (Jones 2022). Among them, male and female factors each account for approximately 20%–30%, while factors from both sides account for about 20%–40%. Additionally, about 10%–20% of causes remain unclear (Stephanie 2021). Successful fertilization is the basic prerequisite for human reproduction, requiring mature sperm to fuse with the oocyte to form a diploid zygote, which can then further develop into a mature individual (Yan et al. 2020). The rapid development of assisted reproductive technology (ART) and the widespread application of genetic testing have enabled many infertile couples to achieve clinical pregnancy and deliver healthy offspring. Among various ART techniques, intracytoplasmic sperm injection (ICSI) is currently the most widely used, especially in patients with severe oligospermia, asthenospermia, teratospermia, or non‐obstructive azoospermia (Eisenberg et al. 2023), and is considered the gold standard for treating male infertility. Although ICSI is effective for many infertile men, 1%–3% of patients still experience repeated fertilization failure even if the concentration, motility, and morphology of their sperm are within normal reference ranges during the ICSI cycle (Sfontouris et al. 2015).

Currently, the mechanisms underlying fertilization failure remain incompletely understood (Hanna et al. 2002). From an etiological perspective, the most common cause of fertilization failure is oocyte activation failure, defined as the inability of the oocyte to resume meiosis and form pronuclei (PN) (Campos et al. 2023) From a clinical outcome perspective, the most common manifestations after fertilization failure are abnormal fertilization (e.g., polyspermy or monopronuclear zygotes) and zygote developmental arrest, i.e., failure of the zygote to undergo normal cleavage and development (Torra‐Massana et al. 2023). With advances in molecular biology techniques, accumulating evidence indicates that fertilization failure is closely related to genetic defects in sperm, especially mutations in key genes regulating oocyte activation, which can lead to oocyte activation deficiency (Qiao et al. 2020). In recent years, multiple genes (e.g., *PLCZ1*, *ACTL7A*, *ACTL9*) have been found to be involved in regulating sperm motility, capacitation, acrosome reaction, and oocyte activation signaling pathways (Barberan et al. 2024; Zhang et al. 2023). In mammalian fertilization, calcium release within the oocyte is a key event, and the frequency, amplitude, and duration of calcium oscillations are species‐specific (Stein et al. 2020). The *PLCZ1* gene encodes the sperm‐specific oocyte activation factor PLCζ protein. After sperm‐oocyte fusion, the sperm releases PLCζ protein into the oocyte cytoplasm, where it hydrolyzes phosphatidylinositol 4,5‐bisphosphate (PIP2), thereby triggering intracellular calcium oscillations, resuming meiosis, and completing fertilization (Hanna et al. 2002). Loss‐of‐function mutations in the *PLCZ1* gene can lead to absent or abnormal PLCζ protein expression, resulting in insufficient or aberrant calcium release and consequently causing oocyte fertilization failure. Clinically, this manifests as fertilization failure after ICSI (with no observable male and female pronuclei, i. e., two pronuclei, 2PN), low fertilization rates, or polyspermy (≥ 3PN) (Kashir et al. 2012; Tong et al. 2024). Multiple studies have shown that assisted oocyte activation (AOA) techniques (e. g., calcium ionophore A23187 or ionomycin) can artificially increase the calcium concentration in the oocyte cytoplasm, effectively rescuing fertilization failure caused by the above mechanisms, and are particularly suitable for patients with specific defects in calcium dynamics during fertilization (Cardona Barberán et al. 2024; Zhao et al. 2023).

The reported cases of *PLCZ1* gene mutations worldwide are mostly associated with compound heterozygous or homozygous mutations, which can account for approximately 30% of ICSI failures caused by male factors, providing a theoretical basis for effective intervention in the reproductive outcomes of such patients (Qiao et al. 2020). However, the clinical phenotypes and molecular mechanisms of new mutations still lack systematic investigation. It is notable that even with ICSI technology, the fertilization rate of patients with *PLCZ1* defects remains significantly lower than normal and is often accompanied by reduced embryonic developmental potential (Kashir et al. 2012). Although AOA can simulate physiological calcium oscillations and partially improve fertilization outcomes, for some mutations, fertilization and embryonic development potential cannot be fully restored, suggesting that genotype–phenotype correlation and mutation specificity may affect intervention efficacy (Tong et al. 2024).

This study conducted clinical, phenotypic, genetic, and bioinformatic analyses of two Chinese male infertility patients with novel homozygous frameshift mutations in *PLCZ1*, aiming to clarify their diagnosis and molecular genetic etiology. Additionally, a literature review of reported national and international cases was performed to systematically summarize the mutation spectrum and clinical phenotypic characteristics of the *PLCZ1* gene. The study also highlights the potential benefits of AOA, providing a theoretical reference for the precise diagnosis and targeted treatment of male infertility.

## 2 Materials and Methods

### 2.1 Collection of Clinical Data and ART Treatment

#### 2.1.1 Participants

Between January 2022 and December 2024, a total of 9236 couples received ART treatment at our center. Among them, 93 cycles exhibited complete fertilization failure or an abnormal fertilization rate ≥ 50% during their first ART cycle. From these, we selected cases meeting the following criteria: (1) female partner with essentially normal ovarian function and ≥ 5 oocytes retrieved; (2) the male partner does not have severe oligospermia, asthenospermia, or teratozoospermia. (i.e., sperm concentration, motility, and morphology did not severely compromise ICSI outcomes); (3) female‐derived factors (e.g., oocyte activation deficiency, zona pellucida abnormalities) excluded based on morphology and previous fertilization history; (4) both partners had a normal karyotype. Among the 93 couples who met these criteria, whole‐exome sequencing (WES) was recommended to search for potential genetic etiologies. Ultimately, two male patients were found to carry homozygous pathogenic frameshift variants in *PLCZ1* (both previously unreported), and their spouses carried no pathogenic variants related to fertilization failure. These two patients were identified as typical cases from a prospective genetic screening of all ICSI fertilization failure cases after excluding other known causes. Because fertilization failure may arise from defects in either sperm or oocytes, every couple included in this study underwent genetic screening and clinical evaluation of both partners to identify the underlying cause and guide subsequent intervention. This study was approved by the Medical Ethics Committee of Gansu Provincial Maternity and Child‐care Hospital (Ethical Review Number: 2023GSFYLS78). All participating couples signed written informed consent forms, clearly understanding the research purpose, potential risks, and data confidentiality terms, and voluntarily agreed to use their clinical data for scientific analysis, which complies with the ethical principles of the Helsinki Declaration.

#### 2.1.2 Clinical Characteristics

Family 1: The couple had been married without contraception and had not conceived for 6 years. They visited our center in May 2022. The female was 31 years old and showed hysterosalpingography results of bilateral partial tubal obstruction, with a tortuous left fallopian tube and a distal reflex near the uterine cornu. Gynecological examination and sex hormone levels were normal. The male was 33 years old; semen analysis showed volume 3.1 mL, sperm concentration 16.45 × 10^6^/mL, progressive motility (PR) 31.67%, and normal morphology 2.87% (reference ≥ 4%, WHO 5th). Both partners had normal karyotypes. The clinical diagnosis was primary infertility, bilateral partial tubal obstruction combined with mild asthenozoospermia.

Family 2: The couple had been trying to conceive for 3 years without success. They visited our center in October 2024. The female was 30 years old; hysterosalpingography showed bilateral partial tubal obstruction, with the left tube tortuous and coiled, and the right tube having an abnormal course. Gynecological examination was unremarkable. The male was 33 years old; semen analysis showed volume 2.3 mL, sperm concentration 87.44 × 10^6^/mL, progressive motility (PR) 6% (PR < 10%, WHO 5th), and normal morphology 4.8%. Both partners had normal karyotypes. The clinical diagnosis was primary infertility due to bilateral partial tubal obstruction combined with severe asthenozoospermia.

#### 2.1.3 Collection of Clinical Data

Clinical data of patients were collected through the hospital information system (HIS), electronic medical record information system (MRIS), and laboratory information management system (LIS). Basic information included the patient's gender, age, presenting complaint, menstrual history, and previous treatment history. Clinical examination and assessment information included: basal hormone levels, thyroid function tests, imaging examinations, immune screening, physical examinations of both spouses, karyotype analysis, and semen analysis results of the male partner. Treatment cycle information includes: ovulation induction protocols, laboratory data, and embryo transfer strategies. These data were used to comprehensively evaluate the fertility status of both couples and to provide a basis for developing individualized assisted reproductive treatment strategies.

#### 2.1.4 ART Treatment

This study was based on the individual characteristics of patients (age, body mass index, basal hormone levels, and ovarian reserve function assessment indicators) and followed the reproductive endocrine regulatory rules to formulate precise ovulation induction plans. Through standardized laboratory procedures, oocytes were obtained. Semen samples were processed using density gradient centrifugation combined with the swim‐up method. ICSI was used for gamete manipulation. In cases where a pathogenic *PLCZ1* variant was identified by genetic testing, AOA using a calcium ionophore was applied during the subsequent ICSI cycle to overcome fertilization failure. The in vitro embryo culture system used sequential media, and dynamic monitoring of embryo development was conducted in a time‐lapse incubator. Embryo quality was evaluated, and high‐quality embryos were prioritized for transfer.

#### 2.1.5 Sample Collection and DNA Extraction

Genomic DNA was extracted from 5 mL of peripheral blood using the Mieji Universal DNA Extraction Kit (batch No. DLI07‐01, Guangzhou Meiji Biotechnology Co. Ltd.) according to the manufacturer's instructions. DNA purity (A260/A280 ratio) and concentration were determined with a NanoDrop 1000 spectrophotometer (Thermo Fisher, USA), yielding concentrations > 20 ng/μL and total amounts > 500 ng for all samples.

#### 2.1.6 WES Sequencing

WES was performed using high‐throughput sequencing (NGS) technology to detect single‐base variations and small fragment insertions/deletions (within 30 bp) in the coding regions and adjacent splicing regions of the entire human genome. It can analyze deletions or duplications of three or more consecutive exons within the tested range. Additionally, for some types of variations for which NGS has limitations, methods such as multiplex ligation‐dependent probe amplification (MLPA), quantitative polymerase chain reaction (qPCR), copy number variation sequencing (CNV‐seq), and gene chips are used for verification. The WES data analysis specifically targeted a curated panel of genes known to be associated with fertilization failure, including both male‐ and female‐related factors. Male‐related genes included *PLCZ1*, *ACTL7A*, *ACTL9*, *DPY19L2*, *SPATA16*, and *PICK1*, among others. Female‐related genes included those involved in oocyte maturation (*TUBB8*, *PATL2*), zona pellucida formation (*ZP1*, *ZP2*, *ZP3*, *ZP4*), oocyte activation deficiency (*WEE2*, *ASTL*), and early embryonic development (*TLE6*, *NLRP5*, *PADI6*, *KHDC3L*). This targeted approach allowed comprehensive screening of both partners to identify the genetic etiology of fertilization failure.

#### 2.1.7 Sanger Sequencing

For the candidate variant sites of the *PLCZ1* (NM_033123.4), specific primers were designed using the online primer design database NCBI Primer‐BLAST (https://www.ncbi.nlm.nih.gov/primer‐blast) (Table S1). DNA fragments containing the variant site regions were amplified by polymerase chain reaction (PCR). The PCR reaction mixture consisted of 12.5 μL of 2 × PCR‐Mix, 10 μL of ddH_2_O, 1.5 μL of DNA, and 0.5 μL each of forward and reverse primers. The reaction conditions were as follows: 4 min of pre‐denaturation at 94°C; 35 cycles of 30 s at 94°C for denaturation, 30 s at 60°C for annealing, and 1 min at 72°C for extension, as follows: 10 min of extension at 72°C; and short‐term storage at 4°C. Sanger sequencing was used to verify the potential pathogenic genes detected by NGS technology. The family members verified for Family 1 were the proband's parents. In this study, the high‐throughput double‐end sequencing primer kit APP‐D (Batch Number: WLC24008, Shenzhen BGI Manufacturing Technology Co. LTD.) was used to convert the linear double‐stranded DNA of the library products into single‐stranded circular DNA libraries compatible with the BGI ZHONGZHI sequencing platform. Then, sequencing was performed in PE150 mode on the DNBSEQ‐T7 sequencing platform (BGI ZHONGZHI Technology Co. Ltd., Shenzhen). Finally, the Sanger sequencing results were compared with the reference sequence of *PLCZ1* (NM_033123.4) using the sequence alignment software SeqMan.

#### 2.1.8 Pathogenicity Analysis of Candidate Variations

Candidate variants were identified using the GATK v3.70 genome analysis toolkit. The HaplotypeCaller module was used to identify candidate variant sites, and the GATK VariantFiltration tool was employed for variant filtering to generate a standard VCF format file. The original data were then compared with the human genome hg19 (GRCh37) reference sequence. The interpretation of candidate pathogenic gene variant sites followed the rating according to the Classification Standards and Guidelines for Genetic Variations of the American College of Medical Genetics and Genomics (ACMG) (Brandt et al. 2020; Richards et al. 2015) (hereinafter referred to as the ACMG guidelines). At the same time, the mutation frequency of the variant sites in the normal population was searched using the human exome database Exome Aggregation Consortium (ExAC) (Lek et al. 2016) (http://exac.broadinstitute.org/) and the genomic variation frequency database Genome Aggregation Database (gnomAD) (Karczewski et al. 2020) (http://gnomad.broadinstitute.org/). The human genetic variation database Human Gene Mutation Database (HGMD) (Stenson et al. 2020) (http://www.hgmd.cf.ac.uk/ac/) was used to query whether the candidate pathogenic gene variation had been previously reported.

#### 2.1.9 Cell Culture, Transfection, and Western Blotting

293T cells were cultured in Dulbecco's Modified Eagle Medium (DMEM) supplemented with 10% fetal bovine serum at 37°C in a 5% CO_2_ incubator. One day before transfection, cells were seeded into 24‐well plates at a density of 2–3 × 10^5^ cells per well. Wild‐type and mutant *PLCZ1* expression vectors (pcDNA3.1(+)‐*PLCZ1*‐WT, pcDNA3.1(+)‐*PLCZ1*‐c.138_139delCA), and pcDNA3.1(+)‐*PLCZ1*‐c.1087del were constructed using seamless cloning technology. Transfection was performed using Lipofectamine 2000 reagent according to the manufacturer's instructions. After 48 h of transfection, cells were harvested and lysed with RIPA buffer containing protease inhibitors. Total protein was separated by (sodium dodecyl sulfate‐polyacrylamide gel electrophoresis) SDS‐PAGE and transferred onto nitrocellulose membranes. The membranes were blocked with 5% non‐fat milk and then incubated with primary antibodies against HA tag (1:3000) and GAPDH (1:60,000), followed by an HRP‐conjugated secondary antibody (1:5000). Protein bands were visualized using enhanced chemiluminescence. The relative protein expression levels were quantified by densitometry and normalized to GAPDH.

### 2.2 Systematic Literature Review

A systematic review was conducted following the PRISMA guidelines (Page et al. 2021). PubMed, CNKI, and Wanfang Database were searched from inception to August 2025 using English terms (“*PLCZ*1”, “Phospholipase C zeta”, “PLC‐zeta”, “PLCzeta”, “PLCζ”, “PLC zeta 1”, “sperm‐specific phospholipase C”, “phospholipase C zeta 1”) and Chinese terms (“*PLCZ1*”, “phospholipase C zeta”, “phospholipase C ζ”, “PLCζ1”, “Sperm‐specific phospholipase C”). We included case reports, case series, and cohort studies that reported male infertility patients with *PLCZ1* mutations and normal oocyte function in their spouses. Exclusion criteria were other genetic abnormalities, non‐genetic infertility, female‐dominant factors, duplicate or incomplete reports, animal/cell studies, and non‐Chinese/non‐English articles. Four trained researchers independently screened titles, abstracts, and full texts, extracted data (first author, year, country, mutation details, clinical phenotype, intervention, and outcomes), and resolved disagreements by discussion with a fifth reviewer.

## 3 Results

### 3.1 Clinical, Genetic, and Functional Characterization of the Two Families

#### 3.1.1 Clinical Data Analysis Results and ART Treatment Results

Family 1: In the first cycle, 29 oocytes were retrieved, among which 27 were at the metaphase II (MII) stage. After ICSI, none of them were fertilized (0PN). Given that both partners had the phenotype of complete fertilization failure and potential genetic factors, it was recommended to conduct WES. In the second cycle, an antagonist protocol was used, with 25 oocytes retrieved, 22 of which were at the MII stage. ICSI fertilization was performed on the MII oocytes, and calcium ionophore A23187 was used to assist oocyte activation. Normal fertilization was achieved in 16 oocytes, high‐quality embryos in 5 oocytes, and 6 blastocysts were formed. Two frozen‐thawed embryos were transplanted, resulting in a single successful pregnancy and the delivery of a healthy male infant (Table 1).

**TABLE 1:** Clinical features of 2 patients during the ART treatment cycle before and after oocyte activation assistance.

Case	Age (Female)	Age (Male)	Years of infertility	Fertilization method	Number of retrieved oocytes	Number of MII oocytes	Normal fertilization number	0PN	≥ 3PN	Number of transplanted embryos	Number of pregnancies	Live birth
1	31	33	6	ICSI	29	27	0	27	0	0	0	0
				ICSI‐AOA	25	22	16	6	6	2	1	1 Boy
2	30	33	3	ICSI	5	3	0	1	2	0	0	0
				ICSI‐AOA	8	5	4	1	0	2	2	2 grils

Abbreviations: MII, metaphase II; PN, pronucleus.

Family 2: In the first cycle, five oocytes were retrieved, among which three were MII oocytes. After ICSI, 1 was not fertilized (0PN), and 2 had abnormal fertilization (3PN). Based on the abnormal fertilization phenotype, it was recommended that the couple undergo WES testing to screen for genetic pathogenic factors. In the second cycle, an antagonist protocol was used, and 8 oocytes were retrieved, including five MII oocytes. ICSI fertilization was performed on the MII oocytes, and ionomycin was used to assist activation. Four normal fertilized oocytes were observed, and 3 high‐quality embryos were formed. During the fresh cycle, we transplanted one 8‐cell grade II embryo and one 7‐cell grade II embryo. The procedure was successful and resulted in the birth of two healthy twin girls (Table 1).

#### 3.1.2 WES And Sanger Sequencing Verification

**FIGURE 1:**
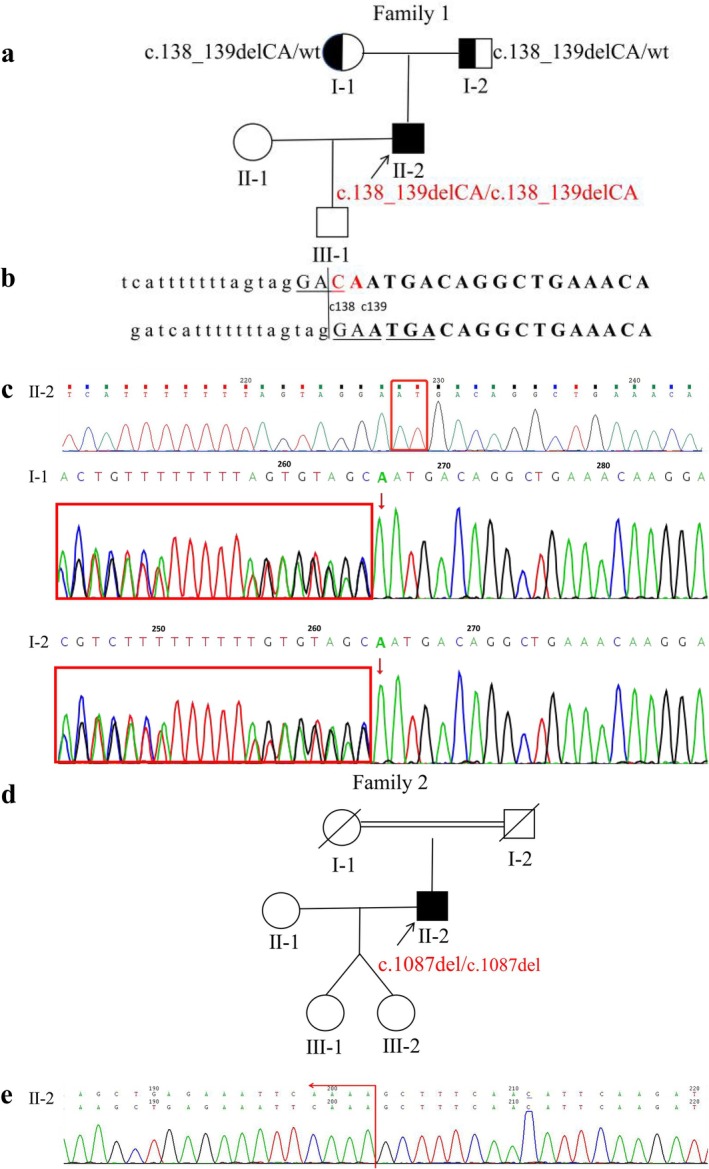
*PLCZ1* mutations in two patients with primary infertility. (a): Pedigree of Family 1; (b): DNA sequence alignment around the mutation site (c.138_139delCA) in Family 1. The upper sequence is the wild‐type sequence, and the lower sequence is the mutant sequence. The deleted “CA” dinucleotide (corresponding to positions c.138_139) is highlighted in red. Nucleotide positions are numbered according to a reference transcript (NM_033123.4); (c): Sanger sequencing chromatogram of Family 1. The arrow indicates the proband, and solid black squares represent affected individuals. (d): Pedigree of Family 2; (e): Sanger sequencing chromatogram of Family 2. The mutation sites are indicated in red.

The WES test revealed that neither of the female partners in the two couples had any pathogenic gene variations related to fertilization disorders or polyspermy, while the male partners both showed gene variations related to fertilization disorders and polyspermy. Specifically, no pathogenic or likely pathogenic variants were identified in any known female‐factor genes associated with oocyte activation deficiency (e. g., *WEE2*, *ASTL*), oocyte maturation (e. g., *TUBB8*, *PATL2*), zona pellucida abnormalities (e. g., *ZP1*–*ZP4*), or early embryonic arrest (e. g., *TLE6*, *NLRP5*, *PADI6*) in either female partner. The sequencing results indicated that each case had a homozygous frameshift mutation in the *PLCZ1*: c.138_139delCA (Family 1) and c.1087del (Family 2). Sanger sequencing validation of the Family 1 pedigree (Figure 1a) showed that the proband's parents (I‐1, I‐2) were both heterozygous carriers (Figure 1c). The DNA sequence alignment around c.138_139delCA (Figure 1b) confirmed the homozygous deletion in the proband and the heterozygous deletion in the parents, indicating that the *PLCZ1* gene variants were inherited from both his father and mother, consistent with autosomal recessive inheritance. Family history inquiry confirmed no consanguinity between the proband's parents. For Family 2 (Figure 1d), familial tracing could not be completed as the male's parents were deceased. The proband of Family 2 has no siblings, and other family members (e. g., uncles, aunts, cousins) were unavailable for testing due to personal reasons and geographical distance. However, further investigation indicated that the proband's parents were reportedly consanguineous, which is marked by a double horizontal line in the pedigree (Figure 1d). This reported consanguinity is consistent with the autosomal recessive inheritance of the homozygous c.1087del variant. Although the variant may theoretically be de novo, suggesting potential gonadal mosaicism or a recessive genetic background, the reported consanguinity provides a plausible explanation for the observed homozygous genotype. Despite the absence of segregation data, the c.1087del variant is classified as pathogenic according to ACMG guidelines (PVS1 + PM2_Supporting + PM3_Supporting). The Sanger sequencing validation of the proband (II‐2) from Family 2 is shown in Figure 1e, confirming the homozygous c.1087del mutation in *PLCZ1*.

#### 3.1.3 Pathogenicity Analysis of the Two Novel Variants

Family 1: The male carries the c.138_139delCA (p. D46Efs*2) variant. This variant leads to a frameshift mutation where aspartic acid at position 46 is replaced by glutamic acid, resulting in the premature formation of a stop codon at position 47. It is predicted to produce a truncated protein containing only 46 amino acids (compared to the normal protein of 608 amino acids). According to the ACMG guidelines, this frameshift variant causes protein truncation exceeding 10% and is located in a critical functional domain of the pathogenic gene (upstream of the EF‐hand). Based on the PVS1 criterion, this variant is highly likely to trigger nonsense‐mediated mRNA decay (NMD) or lead to a complete loss of protein function. The variant is rare and is not recorded in the gnomAD East Asian general population database (PM2_Supporting). Additionally, the patient carries a homozygous variant, consistent with an autosomal recessive inheritance pattern (PM3_Supporting). Integrating the above evidence, this variant is determined to be pathogenic (PVS1 + PM2_Supporting + PM3_Supporting) and is highly correlated with the clinical phenotype of male infertility.

**FIGURE 2:**
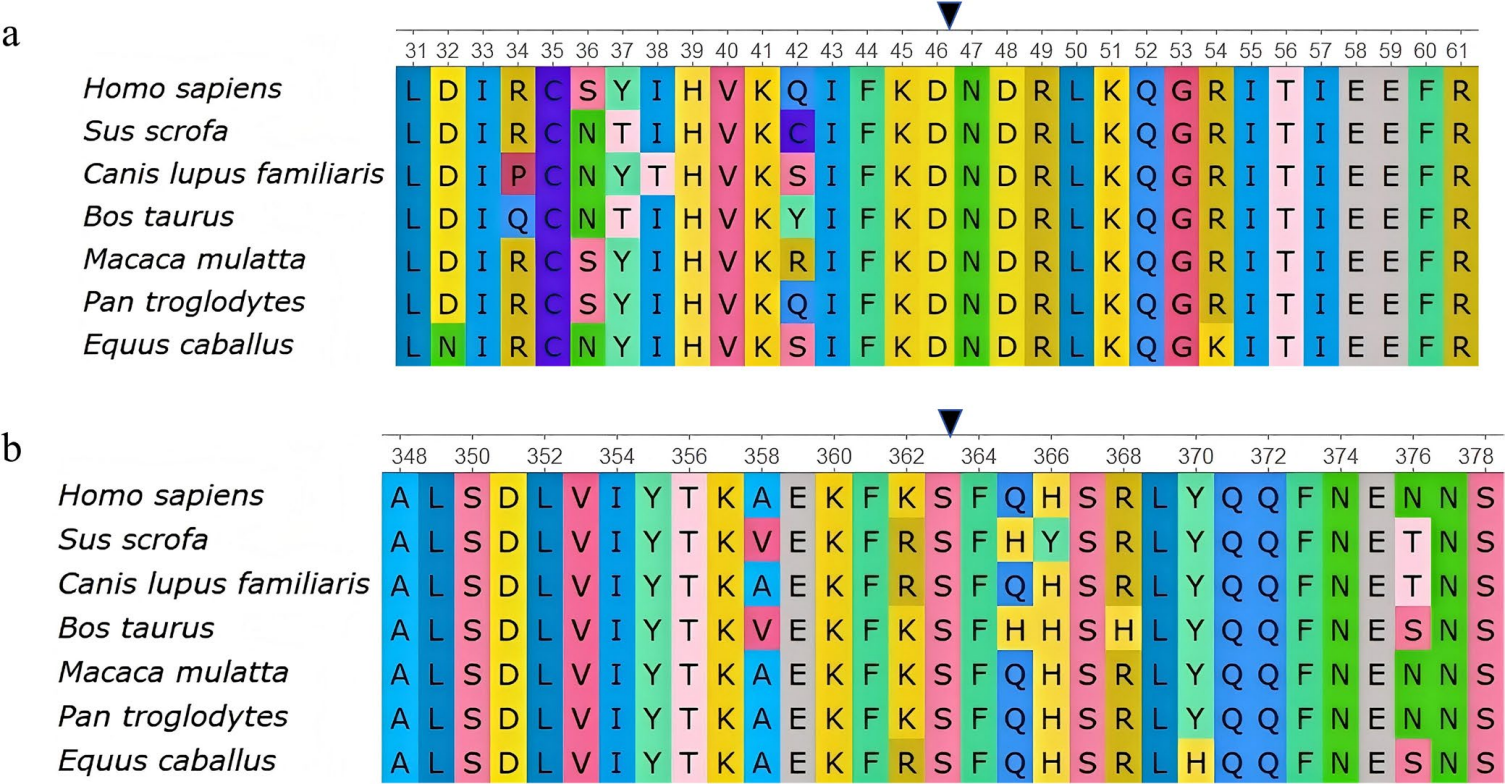
(a): C.138_139delCA (p. D46Efs*2) variant site conservation analysis among different species (the 46th aspartic acid is highly conserved among different species); (b): C.1087del (p. S363Afs*64) variant site conservation analysis among different species (the serine at position 363 is highly conserved among different species, as indicated by the black arrow).

**FIGURE 3:**
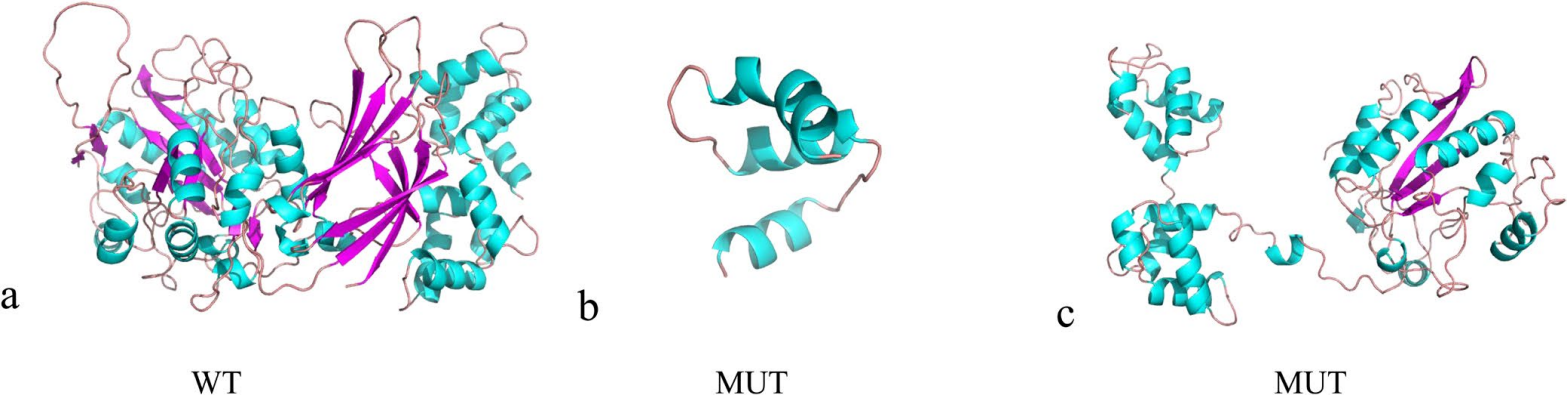
Comparison of amino acid substitution mutation sites in the three‐dimensional structures of wild‐type and variant *PLCZ1* proteins. (a): Wild‐type *PLCZ1* protein; (b): C.138_139delCA (p. D46Efs*2) protein; (c): C.1087del (p. S363Afs*64) protein.

Family 2: The male carries the c.1087del (p. S363Afs*64) variant. This variant leads to a frameshift mutation where serine at position 363 is replaced by alanine, resulting in premature termination at position 427. It is predicted to produce a truncated protein. According to the ACMG guidelines, the evidence for its pathogenicity is as follows: One of the pathogenic mechanisms of the *PLCZ1* gene is loss of function. The detected variant is a frameshift mutation, which may lead to the loss of the catalytic domain (Y core region), significantly impairing PIP_2_ hydrolysis activity. This could result in a loss of more than 10% of amino acids, potentially triggering NMD and rendering the protein nonfunctional (PVSF1). The variant is rare and is not recorded in the gnomAD East Asian general population database (PM2_Supporting). Additionally, the detected variant in this case is homozygous (PM3_Supporting). Integrating the above evidence, this variant is also determined to be pathogenic (PVS1 + PM2_Supporting + PM3_Supporting) and is highly correlated with the clinical phenotype of male infertility. According to bioinformatics protein function software analysis, the variant sites involved in the two cases are located upstream of the EF‐hand domain and within the Y catalytic domain, respectively. The affected aspartic acid at position 46 (Figure 2a) and serine at position 363 (Figure 2b) are highly conserved across different species. Protein three‐dimensional structure diagrams indicate a lack of protein expression, particularly with the c.138_139delCA (p. D46Efs*2) homozygous mutation, which leads to a more extensive loss and severely disrupts protein conformation (Figure 3b). The c.1087del (p. S363Afs*64) homozygous mutation may result in reduced catalytic activity, ultimately affecting protein function (Figure 3c).

#### 3.1.4 Western Blot Validation of Protein Expression

**FIGURE 4:**
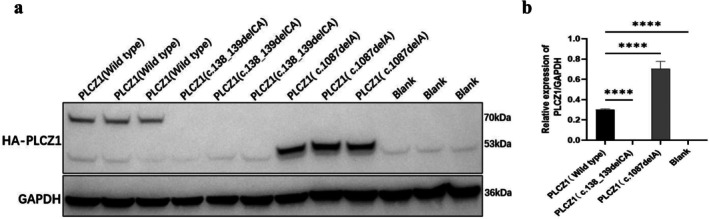
Effect of *PLCZ1* mutations on protein expression in transfected 293T cells. (a): Western blot analysis of *PLCZ1* protein expression in 293T cells transfected with wild‐type (WT) or mutant *PLCZ1* (c.138_139delCA and c.1087del). GAPDH served as a loading control. (b): Densitometric quantification of *PLCZ1* protein levels normalized to GAPDH. Data are shown as mean ± SD (*n* = 3). ***p < 0.001.

To further investigate the impact of the c.138_139delCA and c.1087del (p. S363Afs*64) mutations on *PLCZ1* protein expression, we performed Western blot analysis on 293 T cells transfected with wild‐type or mutant *PLCZ1* constructs. As shown in Figure 4a, the expression level of *PLCZ1* protein was markedly reduced in cells expressing the c.138_139delCA and c.1087del (p. S363Afs*64) mutant compared with those expressing wild‐type *PLCZ1*. Densitometric quantification confirmed a significant decrease in the mutants protein level (*p* < 0.001, Figure 4b). These results indicate that the c.138_139delCA and c.1087del (p. S363Afs*64) variants primarily impair *PLCZ1* protein expression, likely due to reduced protein stability or inefficient translation, leading to a loss of functional PLCζ protein.

### 3.2 Systematic Literature Review

#### 3.2.1 Literature Screening Process and Results

**FIGURE 5:**
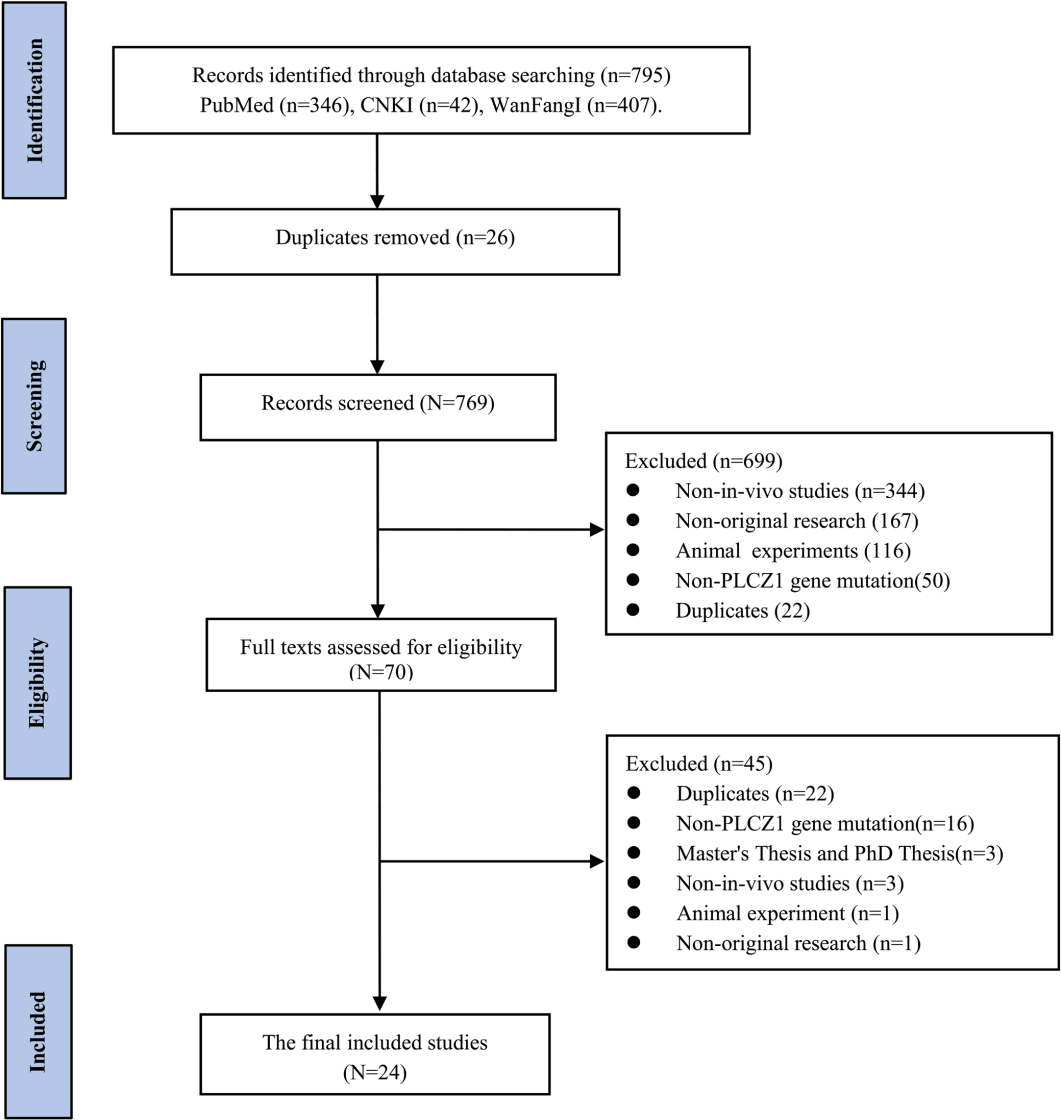
Literature screening process and results.

A total of 795 articles were initially retrieved, and after excluding 26 duplicates, 769 articles underwent title and abstract screening. Following this, 699 articles were excluded, leaving 70 potentially eligible articles for full‐text review. After full‐text assessment, 22 duplicate studies, 16 studies not related to *PLCZ1* gene mutations, 3 in vitro studies, 3 research papers, 1 animal study, and 1 review were excluded. Finally, 24 clinical studies meeting the criteria were included. The PRISMA flow diagram is shown in Figure 5.

#### 3.2.2 Variation Characteristics and Clinical Features of the *PLCZ1* Gene From the Literature

**FIGURE 6:**
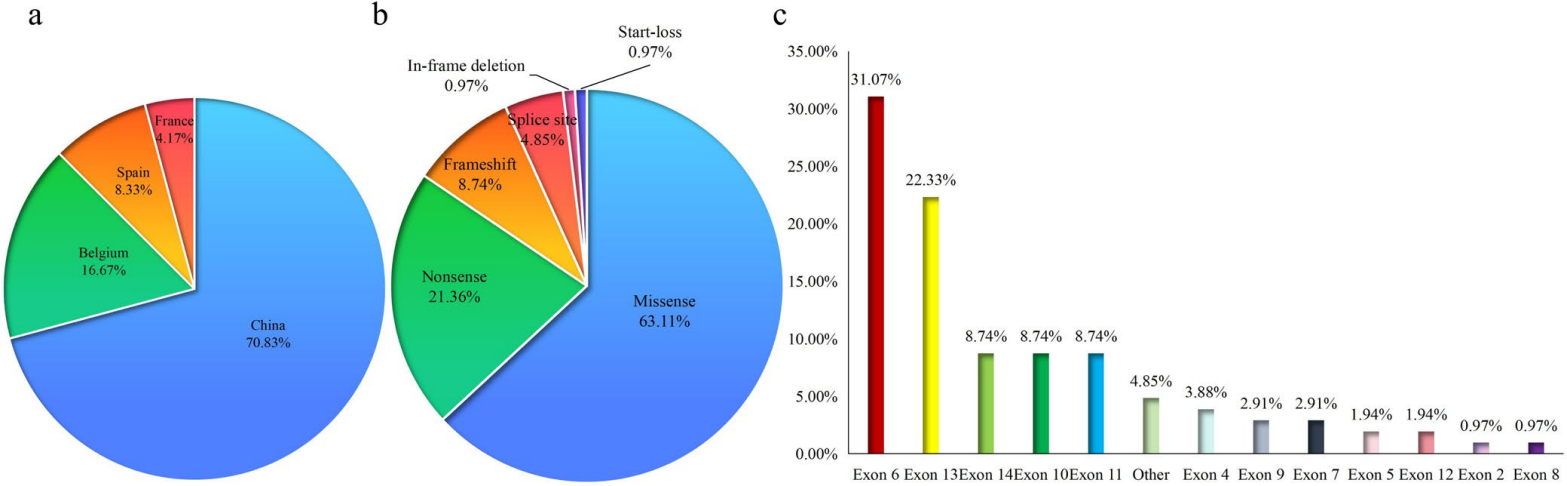
(a) Geographic distribution of *PLCZ*1 mutations; (b) Frequency of mutation types; (c) Exon‐wise distribution.

According to the PRISMA process, a total of 24 studies were finally included for systematic review and analysis, comprising 77 patients (including homozygous, heterozygous, and compound heterozygous individuals; brothers were counted as separate patients). These studies were published between 2009 and 2024, with an increase in the number of studies after 2016. The cases mainly originated from several countries: China (70.83%, 17/24), Belgium (16.67%, 4/24), Spain (8.33%, 2/24), and France (4.17%, 1/24) (Figure 6a). All studies clearly described the detailed information of the patients' gene mutations (cDNA and amino acid changes) (Table S2), and most patients received multiple assisted reproductive technology treatments (Table S3).

A total of 46 distinct cDNA alterations were identified at the nucleotide level (the same mutation was not counted repeatedly across different patients). The overall allele count was 103. The most common mutation overall was c.1499C > T (p. S500L), accounting for 18.4% (19/103) of all mutant alleles, followed by c.588C > A (p. C196X) at 17.4% (18/103). However, when stratified by geographic region, distinct patterns emerged: in the Chinese population, the most frequent mutation was c.588C > A (p. C196X), accounting for 28.6% (18/63) of alleles from Chinese patients, whereas in the European population, the most frequent mutation was c.1499C > T (p. S500L), accounting for 47.5% (19/40) of alleles from European patients. The most frequent mutation type was missense mutation, accounting for 63.11% (65/103) of all mutant alleles, followed by a nonsense mutation at 21.36% (22/103) (Figure 6b). exon 6 was the mutational hotspot, representing 31.07% (32/103) of all mutant alleles, followed by Exon 13 at 22.33% (23/103) (Figure 6c). Regarding the zygotic state distribution among the 77 patients, compound heterozygous and heterozygous mutations each accounted for the highest proportion at 33.7% (26/77), while homozygous mutations accounted for 32.4% (25/77).

**FIGURE 7:**
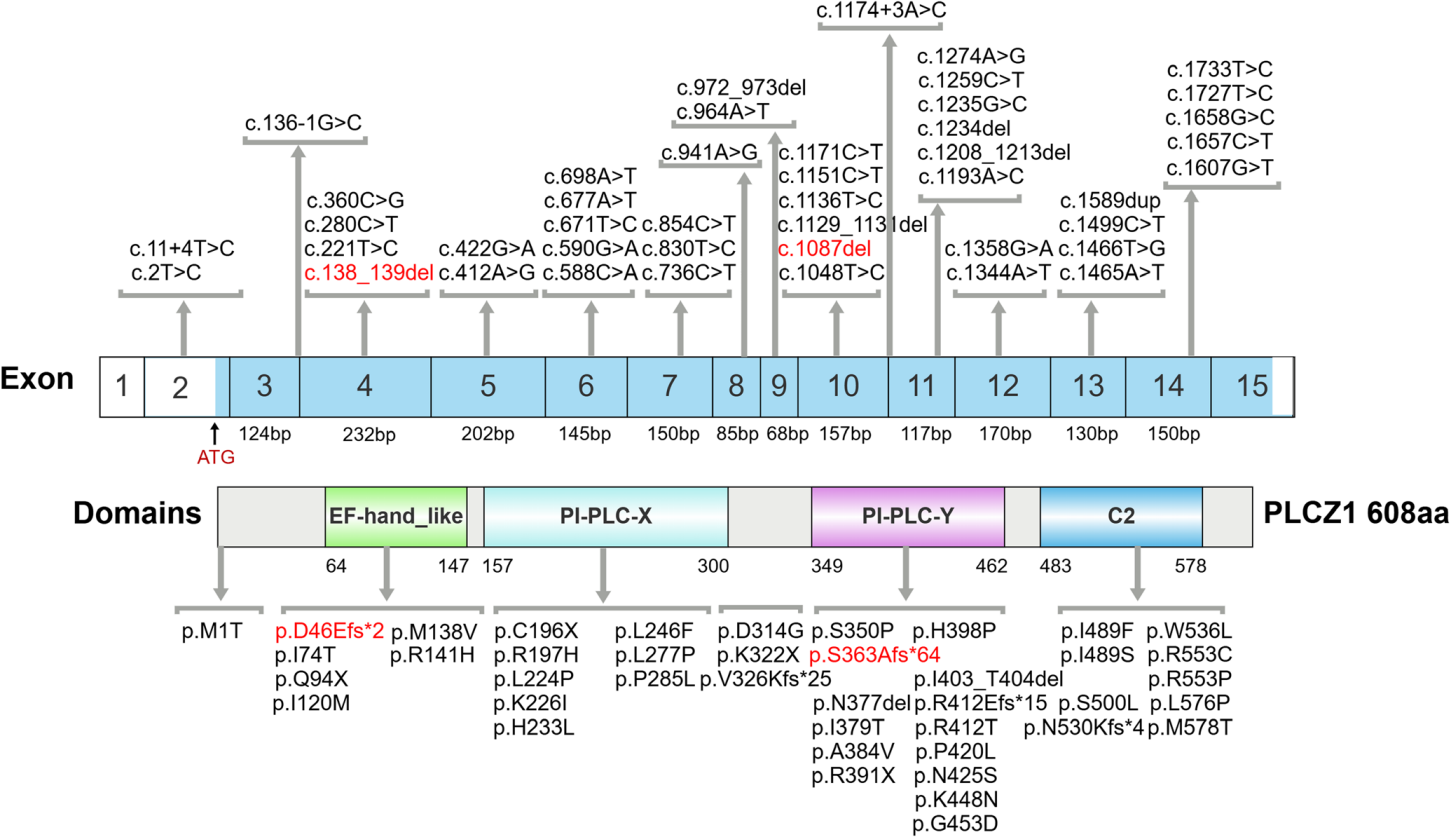
Distribution of *PLCZ*1 variants in exons and protein domains. Data were derived from the 24 studies included in our systematic review (detailed variant‐to‐source mapping in Table S2). Blue bands: Coding regions. Red: Novel variants from this study (c.138_139delCA and c.1087del).

Clinical features: Different variant sites of the *PLCZ1* gene are all associated with male factor infertility. For patients undergoing ART treatment, in most cases, the normal fertilization rate after using ICSI alone is extremely low (mostly 0), thus there are no embryos suitable for transplantation. After the use of activators, the normal fertilization rate significantly improves, increasing from 0 in most cases to 30%–100%, and clinical pregnancy is successfully achieved. There is no significant difference in outcome indicators among different variant sites. The success rate of AOA seems not to be affected by specific variant sites, but more depends on the application of AOA. Currently, the commonly used oocyte activators are ionomycin (concentration 10 μmol/L) and A23187 (concentration 5–10 μmol/L), with an activation time range of 5–15 minutes. Some studies use two treatments (with an interval of 30 min). For semen parameters, the majority of semen volumes are between 1.5–4.0 mL, and the concentration range is wide (14.6–277.3 × 10^6/mL). Some patients have normal concentrations, but their fertilization ability is still affected. The proportion of progressive motility sperm ranges from 20% to 82.7%. The variations c.138_139delCA (p. D46Efs*2) and c.1087del (p.S363Afs*64) discovered in this study are located in the EF‐hand‐like and PI‐PLC‐Y domains of the protein structure. The detailed contents of all studies can be found in Figure 7 and Table S2.

## 4 Discussion

Our study identifies two novel homozygous frameshift mutations in the *PLCZ1* gene, c.138_139delCA (p. D46Efs*2) and c.1087del (p. S363Afs*64), as genetic causes of ICSI failure in two Chinese men. *PLCZ1* is a pathogenic gene responsible for male infertility, located in the 12p12.3 region of chromosome 12, with a molecular weight of approximately 70 kDa. It consists of 15 exons, and NM_033123.4 is its most predominant, conserved, and highly expressed transcript. This gene encodes a phospholipase C protein (608 amino acids), which is located in the acrosome of sperm cells and plays a role in activating fertilization (Alessandra 2024; Saunders et al. 2002). Homozygous or compound heterozygous pathogenic variants of the *PLCZ1* gene can lead to Spermatogenic Failure 17 (MIM#617214), which follows an autosomal recessive inheritance pattern. *PLCZ1* gene variants exhibit significant genetic heterogeneity, primarily in terms of the types and locations of the variants. However, the common clinical phenotype is fertilization failure following ICSI, and ICSI combined with AOA is a consistent and effective intervention strategy. Up to now, a systematic review of the literature has identified multiple reported cases, with missense mutations being the most common type, followed by nonsense mutations, and with exon 6 being a mutational hotspot. Furthermore, significant differences in common variants exist among different geographical and ethnic populations (Figure 6a). The two cases in this study are both homozygous frameshift variants c.138_139delCA (p. D46Efs*2) and c.1087del (p. S363Afs*64), located in exons 4 and 10 respectively, which are new variants discovered in Chinese male infertility patients. This expands the pathogenic variant spectrum of the *PLCZ1*. In Family 1, no parental consanguinity was reported, consistent with autosomal recessive inheritance from heterozygous carrier parents. In Family 2, reported parental consanguinity further supports the autosomal recessive inheritance of the homozygous *PLCZ1* variant. Combined with clinical phenotypes and in vitro experimental validation, our findings reveal the molecular mechanism by which these mutations disrupt *PLCZ1* protein function: the c.138_139delCA mutation leads to complete loss of detectable PLCζ protein, while the c.1087del mutation results in a severely truncated protein with markedly reduced expression, both ultimately leading to fertilization failure. These data provide new experimental evidence for genetic diagnosis and precise intervention in male infertility.

Oocyte activation is the core process of mammalian fertilization, and its molecular mechanism relies on the Ca^2+^ oscillation signal mediated by PLCZ1. As the “molecular switch” that triggers embryonic development, PLCZ1 hydrolyzes phosphatidylinositol 4, 5‐bisphosphate (PIP_2_) to generate inositol triphosphate (IP₃), inducing Ca^2+^ release within the oocyte, thereby completing the initiation of fertilization (Swann and Lai 2016; Yu et al. 2012). The two variants identified in this study are both located in the key functional domain of PLCZ1. The decrease in enzyme activity caused by the two novel mutations is strongly supported by our in vitro protein expression data (Figure 4b), which demonstrate complete loss (c.138_139delCA) or severe truncation (c.1087del) of PLCζ protein, indicating loss‐of‐function. The c.138_139delCA (p. D46Efs2) variant is situated upstream of the N‐terminal EF‐hand domain of the PLCζ protein, causing a frameshift at aspartic acid 46 and premature termination codon formation. Notably, a frameshift mutation not only affects the domain in which it occurs but also alters the reading frame of all downstream amino acid sequences, often introducing a premature stop codon. For the c.138_139delCA mutation, the stop codon occurs at position 47, resulting in a truncated protein of only 46 amino acids. This leads to the complete loss of almost all functional domains of PLCζ, including the C2 domain, the catalytic X and Y *domains* (which form the catalytic core), and the C‐terminal EF‐hand motif (Lee et al. 2025). Consequently, this mutation abolishes not only the Ca^2+^‐binding ability of the N‐terminal EF‐hand but also the phospholipase catalytic activity and membrane‐targeting capacity. Similarly, the c.1087del (p. S363Afs64) variant is located in exon 10, causing a frameshift at serine 363 within the catalytic domain and extending the protein to 427 amino acids. Although this mutant retains a portion of the catalytic X domain, the frameshift disrupts the catalytic Y domain and the C‐terminal EF‐hand motif, both of which are essential for enzymatic activity and Ca^2+^‐dependent regulation. Thus, both frameshift mutations ultimately result in a complete loss of functional PLCζ protein by affecting multiple key domains (EF‐hand, C2, X, Y), leading to fertilization failure. The catalytic domain (X/Y catalytic core) is critical for PLCζ to hydrolyze phosphatidylinositol 4,5‐bisphosphate (PIP_2_) and generate inositol trisphosphate (IP₃). These variants may disrupt the integrity of the catalytic pocket, significantly reducing or abolishing enzymatic activity, ultimately leading to the absence of Ca^2+^ oscillations.

The two infertile men presented with heterogeneous semen parameters, but both consistently resulted in fertilization failure after ICSI Family 1 exhibited teratozoospermia (normal morphology rate < 4%), with no fertilization (0PN) observed in all 27 MII oocytes after ICSI. Family 2 showed severe asthenozoospermia (progressive motility < 10%), with 2 out of 3 MII oocytes abnormally fertilized (3PN) and 1 unfertilized (0PN) after ICSI. It is noteworthy that abnormal fertilization (3PN) may be associated with aberrant localization and expression of PLCζ protein in sperm (Tong et al. 2024). These findings suggest that complete loss of *PLCZ1* function leads to total fertilization failure, while partial functional impairment may result in abnormal fertilization. This phenomenon requires validation in larger cohort studies. Notably, conventional ICSI cannot resolve such issues, as the technique only delivers the sperm genome without compensating for the deficiency of PLCζ protein. For fertilization barriers caused by *PLCZ1* defects, AOA (e.g., calcium ionophores A23187 or ionomycin) effectively simulates physiological Ca^2+^ oscillations, successfully reversing fertility outcomes. Subsequently, our team applied calcium ionophore A23187 activation for Family 1, achieving a normal fertilization rate of 72.7% (16/22), resulting in high‐quality blastocysts and the birth of a healthy offspring. For Family 2, ionomycin activation yielded a normal fertilization rate of 80.0% (4/5), leading to the successful birth of twins. These results confirm that AOA can compensate for *PLCZ1* functional deficiency through exogenous Ca^2+^ signaling, providing an effective solution for such patients. Additionally, microinjection of *PLCZ1* mRNA into MII oocytes can induce Ca^2+^ oscillations similar to those during fertilization, though its safety remains unclear (Takashi 2017). Alternatively, microinjection of recombinant PLCζ protein may be a feasible strategy to rescue PLCζ deficiency if performed immediately after ICSI fertilization failure. However, further research is needed to develop safe and stable purified protein molecules, and thus, it has not yet been implemented clinically (Gat and Orvieto 2017; Kuroda et al. 2018).

## 5 Conclusion

In this study, two novel homozygous frameshift mutations in PLCZ1 were identified as the cause of fertilization failure in two couples, thereby expanding the spectrum of *PLCZ*1 gene variants associated with male infertility. Following ICSI combined with assisted oocyte activation, both patients achieved successful live births. While a literature review has revealed various *PLCZ*1 mutations, further research is still needed to elucidate their transcriptional and translational regulation. A limitation of this study is the absence of independent wild‐type controls; future studies using gene‐edited animal models may further clarify the underlying mechanisms.

## Author Contributions

Jinwei Yang, Bo Yan, and Zhiqiang Wang mainly contributed to the study design, data analysis, and manuscript writing. Zhizhuo Wei, Zijing Niu, and Huanjing Wang collated the patients' samples. Tongguang Wang, Yan Wei, Yue Da, Lin Zhang, and Fangzhu Wang performed the systematic review. Jinwei Yang and Wei Jiang conducted the manuscript writing with assistance from all authors. Yali Ni and Zhiqiang Wang conceived the study and supervised its progress. All authors read and approved the final manuscript.

## Funding

This work was supported by the Natural Science Foundation of Gansu Province, 23JRRA1389. Key Research and Development Program of Gansu Province, 25YFFA056. Science and Technology Program of Gansu Province, 21JR7RA679.

## Ethics Statement

This study was approved by the Ethics Committee of Gansu Provincial Maternity and Child‐care Hospital. Written informed consent was obtained from the families.

## Conflicts of Interest

The authors declare no conflicts of interest.

## Supporting information


**Table S1:** Sanger sequencing primers for *PLCZ1* gene validation.

**Table S2:** Basic characteristics of included studies on *PLCZ1* mutations.

**Table S3:** Baseline characteristics and treatment outcomes of patients with *PLCZ1* mutations.

## Data Availability

The data that support the findings of this study are available on request from the corresponding author. The data are not publicly available due to privacy or ethical restrictions.
