# Supplementary material for: Clinical Features and PLCZ1 Gene Variants in Two Cases of Male Infertility: A Case Series and Literature Review
Source: Mol Genet Genomic Med. 2026 Jun 15;14(6):e70250. doi: 10.1002/mgg3.70250 (PMC13269656; doi:10.1002/mgg3.70250)
Supplement: Supplementary file 1 — Table S1: Sanger sequencing primers for PLCZ1 gene validation. [file MGG3-14-e70250-s002.docx]

**Table S1** Sanger sequencing primers for the *PLCZ1* gene

| Family | Variant site | Forward primer sequence（5’-3’） | Reverse primer sequence（5’-3’） | Product length（bp） |
| --- | --- | --- | --- | --- |
| 1 | c.138_139delCA(p.D46Efs*2) | ACCTGGAATCCTCATTTTCTCCCA | ACATACCTTCTTCGATAGGCTCGT | 490 |
| 2 | c.1087del(p.S363Afs*64) | TGGTGTTTGAGGAGATAATCTTATG | CTGAGGTATGTAACCCCATTCTAA | 414 |
