# Supplementary material for: Clinical Features and PLCZ1 Gene Variants in Two Cases of Male Infertility: A Case Series and Literature Review
Source: Mol Genet Genomic Med. 2026 Jun 15;14(6):e70250. doi: 10.1002/mgg3.70250 (PMC13269656; doi:10.1002/mgg3.70250)
Supplement: Supplementary file 2 — Table S2: Basic characteristics of included studies on PLCZ1 mutations. [file MGG3-14-e70250-s003.docx]

**Table S2** Basic characteristics of included studies on *PLCZ1* mutations

| Author (year) | Country | Patient ID | cDNA alteration | Amino acid alteration | Zygotic state | Mutation type | Exon/intron |
| --- | --- | --- | --- | --- | --- | --- | --- |
| (Heytens et al., 2009) | Belgium |  | c.1193A>C | p.H398P | Heterozygous | Missense | Exon 11 |
| (Escoffier et al., 2016)^*^ | France | P1 | c.1465A>T | p.I489F | Homozygous | Missense | Exon 13 |
|  |  | P2 | c.1465A>T | p.I489F | Homozygous | Missense | Exon 13 |
| (Ferrer-Vaquer et al., 2016) | Spain | P1 | c.698A>T | p.H233L | Heterozygous | Missense | Exon 6 |
|  |  | P2 | c.854C>T | p.R197H | Heterozygous | Missense | Exon 7 |
|  |  | P3 | c.1499C>T | p.S500L | Heterozygous | Missense | Exon 13 |
| (Torra-Massana et al., 2019) | Spain | P1 | c.590G>A  c.1499C>T | p.R197H  p.S500 L | Compound heterozygous | Missense  Missense | Exon 6  Exon 13 |
|  |  | P2 | c.698A>T | p.H233L | Heterozygous | Missense | Exon 6 |
|  |  | P3 | c.972_973delAG | p.V326K fs*25 | Heterozygous | Frameshift | Exon 9 |
|  |  | P4 | c.1499C>T | p.S500L | Heterozygous | Missense | Exon 13 |
|  |  | P5 | c.1499C>T | p.S500L | Heterozygous | Missense | Exon 13 |
|  |  | P6 | c.671T>C | p.L224P | Heterozygous | Missense | Exon 6 |
|  |  | P7 | c.1499C>T | p.S500L | Heterozygous | Missense | Exon 13 |
|  |  | P8 | c.1499C>T | p.S500L | Heterozygous | Missense | Exon 13 |
|  |  | P9 | c.1499C>T | p.S500L | Homozygous | Missense | Exon 13 |
|  |  | P10 | c.360C>G | p.I120M | Heterozygous | Missense | Exon 4 |
|  |  | P11 | c.1499C>T | p.S500L | Heterozygous | Missense | Exon 13 |
|  |  | P12 | c.1499C>T | p.S500L | Heterozygous | Missense | Exon 13 |
|  |  | P13 | c.1499C>T | p.S500L | Heterozygous | Missense | Exon 13 |
| (Dai et al., 2019) | China | P1 | c.588C>A | p.C196X | Homozygous | Nonsense | Exon 6 |
|  |  | P2 | c.1048T>C | p.S350P | Homozygous | Missense | Exon 10 |
|  |  | P3 | c.736C>T | p.L246F | Homozygous | Missense | Exon 7 |
| (Wang et al., 2020) | China |  | c.588C>A | p.C196X | Homozygous | Nonsense | Exon 6 |
| (Mu et al., 2020) | China | P1 | c.588C>A | p.C196X | Homozygous | Nonsense | Exon 6 |
|  |  | P2 | c.588C>A  c.1259C>T | p.C196X  p.P420L | Compound heterozygous | Nonsense  Missense | Exon 6  Exon 11 |
|  |  | P3 | c.590G>A | p.R197H | Homozygous | Missense | Exon 6 |
|  |  | P4 | c.972_973delAG  c.1234del | p.V326Kfs*25  p.R412Efs*15 | Compound heterozygous | Frameshift  Frameshift | Exon 9  Exon 11 |
| (Yuan et al., 2020) | China | P1 | c.1259C>T  c.1733T>C | p.P420L  p.M578T | Compound heterozygous | Missense  Missense | Exon 11  Exon 14 |
|  |  | P2 | c.1727T>C | p.L576P | Homozygous | Missense | Exon 14 |
| (Yan et al., 2020) | China | P1 | c.588C>A | p.C196X | Homozygous | Nonsense | Exon 6 |
|  |  | P2 | c.588C>A  c.830T>C | p.C196X  p.L277P | Compound heterozygous | Nonsense  Missense | Exon 6  Exon 7 |
|  |  | P3 | c.1129_1131delAAT  c.1733T>C | p.N377del  p.M578T | Compound heterozygous | Missing mutation within the frame  Missense | Exon 10  Exon 14 |
|  |  | P4 | c.1151C>T | p.A384V | Homozygous | Missense | Exon 10 |
|  |  | P5 | c.570+1G>T  c.1344A>T | p.V189Cfs*12  p.K448N | Compound heterozygous | Splice  Missense | The boundary between exon 6 and intron 5  Exon 12 |
| (Yuan et al., 2021) | China |  | c.1658G>C | p.R553P | Homozygous | Missense | Exon 14 |
| (Zhang et al., 2022) | China |  | c.588C>A  c.1589dupA | p.C196X  p.N530Kfs*3 | Compound heterozygous | Nonsense  Frameshift | Exon 6  Exon 13 |
| (Cardona Barberán et al., 2023) | Belgium |  | c.1499C>T | p.S500L | Homozygous | Missense | Exon 13 |
| (Bekaert et al., 2023) | Belgium |  | c.136-1G>C | p.? | Heterozygous | Splice | The boundary between intron 3 and exon 4 |
| (Lin et al., 2023) | China | P1 | c.588C>A  c.1466T>G | p.C196X  p.I489S | Compound heterozygous | Nonsense  Missense | Exon 6  Exon 13 |
|  |  | P2 | c.588C>A  c.1208_1213del | p.C196X  p.I403_404del | Compound heterozygous | Nonsense  Inframe deletion | Exon 6  Exon 11 |
|  |  | P3 | c.1151C>T | p.A384V | Homozygous | Missense | Exon 10 |
|  |  | P4 | c.1607G>T | p.W536X | Homozygous | Nonsense | Exon 14 |
| (Zhao et al., 2023) | China | P1 | c.1174+3A>C  c.1274A>G | p.?  p.N425S | Compound heterozygous | Splice  Missense | Intron 10  Exon 11 |
|  |  | P2 | c.136-1G>C  c.1358G>A | p.?  p.G453D | Compound heterozygous | Splice  Missense | Intron 3  Exon 12 |
| (Peng et al., 2022) | China | P1 | c.588C>A | p.C196X | Homozygous | Nonsense | Exon 6 |
|  |  | P2 | c.2T>C  c.590G>A | p.M1T  p.R197H | Compound heterozygous | Startloss  Missense | Exon 2  Exon 6 |
| (Li et al., 2023) | China |  | c.588C>A | p.C196X | Homozygous | Nonsense | Exon 6 |
| (Wu et al., 2024) | China |  | c.588C>A  c.590G>A | p.C196X  p.R197H | Compound heterozygous | Nonsense  Missense | Exon 6  Exon 6 |
| (Barberan et al., 2024) | Belgium | P1 | c.698A>T  c.964A>T | p.H233L  p.K322X | Compound heterozygous | Missense  Nonsense | Exon 6  Exon 9 |
|  |  | P2 | c.1499C>T | p.S500L | Heterozygous | Missense | Exon 13 |
|  |  | P3 | c.1499C>T | p.S500L | Heterozygous | Missense | Exon 13 |
|  |  | P4 | c.1499C>T | p.S500L | Homozygous | Missense | Exon 13 |
|  |  | P5 | c.422G>A | p.R141H | Heterozygous | Missense | Exon 5 |
|  |  | P6 | c.1499C>T | p.S500L | Heterozygous | Missense | Exon 13 |
|  |  | P7 | c.1499C>T | p.S500L | Homozygous | Missense | Exon 13 |
|  |  | P8 | c.1499C>T | p.S500L | Homozygous | Missense | Exon 13 |
|  |  | P9 | c.698A>T | p.H233L | Heterozygous | Missense | Exon 6 |
|  |  | P10 | c.1499C>T | p.S500L | Homozygous | Missense | Exon 13 |
|  |  | P11 | c.280C>T  c.1499C>T | p.Q94X  p.S500L | Compound heterozygous | Nonsense  Missense | Exon 4  Exon 13 |
|  |  | P12 | c.221T>C | p.I74T | Heterozygous | Missense | Exon 4 |
|  |  | P13 | c.698A>T | p.H233L | Heterozygous | Missense | Exon 6 |
|  |  | P14 | c.698A>T | p.H233L | Heterozygous | Missense | Exon 6 |
|  |  | P15 | c.1136T>C | p.I379T | Heterozygous | Missense | Exon 10 |
|  |  | P16 | c.698A>T | p.H233L | Heterozygous | Missense | Exon 6 |
| (Che et al., 2024) | China | P1 | c.11+4T>C  c.588C>A | p.?  p.C196X | Compound heterozygous | Splice  Nonsense | Intron 2  Exon 6 |
|  |  | P2 | c.588C>A  c.677A>T | p.C196X  p.K226I | Compound heterozygous | Nonsense  Missense | Exon 6  Exon 6 |
| (Tong et al., 2024)^#^ | China | P1 | c.1154C>T  c.1234del | p.R385L  p.R412Efs*15 | Compound heterozygous | Missense  Frameshift deletion | Exon 10  Exon 11 |
|  |  | P2 | c.1154C>T  c.1234del | p.R385L  p.R412Efs*15 | Compound heterozygous | Missense  Frameshift deletion | Exon 10  Exon 11 |
|  |  | P3 | c.1733T>C | p.M578T | Homozygous | Missense | Exon 14 |
| (Li et al., 2024) | China | P1 | c.941A>G  c.588C>A | p.D314G  p.C196X | Compound heterozygous | Missense  Nonsense | Exon 8  Exon 6 |
|  |  | P2 | c.1171C>T  c.588C>A | p.R391X  p.C196X | Compound heterozygous | Nonsense  Nonsense | Exon 10  Exon 6 |
|  |  | P3 | c.1657C>T  c.1733T>C | p.R553C  p.M578T | Compound heterozygous | Missense  Missense | Exon 14  Exon 14 |
|  |  | P4 | c.590G>A  c.1235G>C | p.R197H  p.R412T | Compound heterozygous | Missense  Missense | Exon 6  Exon 11 |
|  |  | P5 | c.588C>A  c.412A>G | p.C196X  p.M138V | Compound heterozygous | Nonsense  Missense | Exon 6  Exon 5 |
| (Hu et al., 2025) | China |  | c.588C>A  c.1733T>C | p.C196X  p.M578T | Compound heterozygous | Nonsense  Missense | Exon 6  Exon 14 |
| (Our study 2025) | China | P1 | c.138_139delCA | p.D46Efs*2 | Homozygous | Frameshift | Exon 4 |
|  |  | P2 | c.1087del | p.S363Afs*64 | Homozygous | Frameshift | Exon 10 |

Notes: ^*^: P1 and P2 in Escoffier et al., 2016 are brothers; ^#^: P1 and P2 in Tong et al., 2024 are brothers; p.?: Splicing site mutation, Unknown protein product.

**REFERENCES**

Barberan, A. C., Guggilla, R. R., Colenbier, C., Van der Velden, E., Rybouchkin, A., Stoop, D., Leybaert, L., Coucke, P., Symoens, S., Boel, A., Meerschaut, F. V., & Heindryckx, B. (2024). High rate of detected variants in male PLCZ1 and ACTL7A genes causing failed fertilization after ICSI. *Human Reproduction Open, 2024*(4). doi:10.1093/hropen/hoae057

Bekaert, B., Boel, A., De Witte, L., Vandenberghe, W., Popovic, M., Stamatiadis, P., Cosemans, G., Tordeurs, L., De Loore, A.-M., Chuva de Sousa Lopes, S. M., De Sutter, P., Stoop, D., Coucke, P., Menten, B., & Heindryckx, B. (2023). Retained chromosomal integrity following CRISPR-Cas9-based mutational correction in human embryos. *Molecular Therapy, 31*(8), 2326-2341. doi:10.1016/j.ymthe.2023.06.013

Cardona Barberán, A., Bonte, D., Boel, A., Thys, V., Paredis, R., Machtelinckx, F., De Sutter, P., De Croo, I., Leybaert, L., Stoop, D., Coucke, P., Vanden Meerschaut, F., & Heindryckx, B. (2023). Assisted oocyte activation does not overcome recurrent embryo developmental problems. *Human Reproduction, 38*(5), 872-885. doi:10.1093/humrep/dead051

Che, J., Wu, H., Zeng, S., Wu, Y., Dai, J., Cheng, D., Gong, F., Lu, G., Lin, G., & Dai, C. (2024). Defects in phospholipase C zeta cause polyspermy and low fertilization after conventional IVF:not just ICSI failure. *The Asian Journal of Menology (English Edition), 26*(2), 175-182.

Dai, J., Dai, C., Guo, J., Zheng, W., Zhang, T., Li, Y., Lu, C., Gong, F., Lu, G., & Lin, G. (2019). Novel homozygous variations in PLCZ1 lead to poor or failed fertilization characterized by abnormal localization patterns of PLCζ in sperm. *Clinical Genetics, 97*(2), 347-351. doi:10.1111/cge.13636

Escoffier, J., Lee, H. C., Yassine, S., Zouari, R., Martinez, G., Karaouzène, T., Coutton, C., Kherraf, Z.-e., Halouani, L., Triki, C., Nef, S., Thierry-Mieg, N., Savinov, S. N., Fissore, R., Ray, P. F., & Arnoult, C. (2016). Homozygous mutation of PLCZ1 leads to defective human oocyte activation and infertility that is not rescued by the WW-binding protein PAWP. *Human Molecular Genetics, 25*(5), 878-891. doi:10.1093/hmg/ddv617

Ferrer-Vaquer, A., Barragan, M., Freour, T., Vernaeve, V., & Vassena, R. (2016). PLCζ sequence, protein levels, and distribution in human sperm do not correlate with semen characteristics and fertilization rates after ICSI. *Journal of Assisted Reproduction & Genetics, 33*(6), 747-756.

Heytens, E., Parrington, J., Coward, K., Young, C., Lambrecht, S., Yoon, S. Y., Fissore, R. A., Hamer, R., Deane, C. M., Ruas, M., Grasa, P., Soleimani, R., Cuvelier, C. A., Gerris, J., Dhont, M., Deforce, D., Leybaert, L., & De Sutter, P. (2009). Reduced amounts and abnormal forms of phospholipase C zeta (PLC) in spermatozoa from infertile men. *Human Reproduction, 24*(10), 2417-2428. doi:10.1093/humrep/dep207

Hu, Y. Y., Wang, Q. Y., Yan, X., Sun, Z. F., Zhang, X., Zhang, Y., & Zhang, C. j. (2025). One case of PLCZ1 compound heterozygous mutation patient underwent ICSI combined with oocyte activation assistance, and a literature review was conducted. *Chinese Journal of Reproduction and Contraception, 45*(6), 618-621. doi:10.3760/cma.j.cn101441-20241105-00405

Li, C. H., Cheng, D. K., Ren, H. Q., & Li, C. Y. (2023). A Case Report of Complete Fertilization Failure Caused by Molecular Genetic Factors. *Journal of Maternal and Child Health, 2*(7), 72-74.

Li, Q., Guo, J. C., Huang, G. L., Wu, N., Chen, S., Dai, J., Zhang, X. G., Zhang, G. H., Zhi, W. W., Yan, J. R., Zheng, R., Yan, F., Yan, Z., Wu, L., Wu, S. X., Ji, Z. L., Zeng, J. Z., Lin, G., Li, B., & Xu, W. M. (2024). Novel PLCZ1 compound heterozygous mutations indicate gene dosage effect involved in total fertilisation failure after ICSI. *Reproduction, 168*(4). doi:10.1530/rep-23-0466

Lin, Y., Huang, Y., Li, B., Zhang, T., Niu, Y., Hu, S., Ding, Y., Yao, G., Wei, Z., Yao, N., Yao, Y., Lu, Y., He, Y., Zhu, Q., Zhang, L., & Sun, Y. (2023). Novel mutations in PLCZ1 lead to early embryonic arrest as a male factor. *Frontiers in Cell and Developmental Biology, 11*. doi:10.3389/fcell.2023.1193248

Mu, J., Zhang, Z., Wu, L., Fu, J., Chen, B., Yan, Z., Li, B., Zhou, Z., Wang, W., Zhao, L., Dong, J., Kuang, Y., Sun, X., He, L., Wang, L., & Sang, Q. (2020). The identification of novel mutations in PLCZ1 responsible for human fertilization failure and a therapeutic intervention by artificial oocyte activation. *Molecular Human Reproduction, 26*(2), 80-87. doi:10.1093/molehr/gaaa003

Peng, Y., Lin, Y., Deng, K., Shen, J., Cui, Y., Liu, J., Yang, X., & Diao, F. (2022). Mutations in PLCZ1 induce male infertility associated with polyspermy and fertilization failure. *Journal of Assisted Reproduction and Genetics, 40*(1), 53-64. doi:10.1007/s10815-022-02670-2

Tong, K., Liu, W., Sun, L., Liu, D., Xiang, Y., Li, C., Chai, L., Chen, K., Huang, G., & Li, J. (2024). Novel PLCZ1 mutation caused polyspermy during in vitro fertilization. *Asian Journal of Andrology, 26*(4), 389-395. doi:10.4103/aja202376

Torra-Massana, M., Cornet-Bartolomé, D., Barragán, M., Durban, M., Ferrer-Vaquer, A., Zambelli, F., Rodriguez, A., Oliva, R., & Vassena, R. (2019). Novel phospholipase C zeta 1 mutations associated with fertilization failures after ICSI. *Human Reproduction, 34*(8), 1494-1504. doi:10.1093/humrep/dez094

Wang, F., Zhang, J., Kong, S., Li, C., Zhang, Z., He, X., Wu, H., Tang, D., Zha, X., Tan, Q., Duan, Z., Cao, Y., & Zhu, F. (2020). A homozygous nonsense mutation of PLCZ1 cause male infertility with oocyte activation deficiency. *Journal of Assisted Reproduction and Genetics, 37*(4), 821-828. doi:10.1007/s10815-020-01719-4

Wu, T. H., Chen, C. M., Sun, Q., Xu, F., & Mo, M. L. (2024). One case of PLCZ1 compound heterozygous mutation leading to low IVF multiple-nucleus fertilization and ICSI fertilization. *Chinese Journal of Eugenics and Genetics, 32*(5), 1034-1039.

Yan, Z., Fan, Y., Wang, F., Yan, Z., Li, M., Ouyang, J., Wu, L., Yin, M., Zhao, J., Kuang, Y., Li, B., & Lyu, Q. (2020). Novel mutations in PLCZ1 cause male infertility due to fertilization failure or poor fertilization. *Human Reproduction, 35*(2), 472-481. doi:10.1093/humrep/dez282

Yuan, P., Yang, C., Ren, Y., Yan, J., Nie, Y., Yan, L., & Qiao, J. (2021). A novel homozygous mutation of phospholipase C zeta leading to defective human oocyte activation and fertilization failure. *Human Reproduction, 35*(4), 977-985. doi:10.1093/HUMREP/DEZ293

Yuan, P., Zheng, L. Y., Liang, H., Lin, Q. Y., Ou, S. B., Zhu, Y. Q., Lai, L. H., Zhang, Q. X., He, Z. Y., & Wang, W. J. (2020). Novel mutations in the PLCZ1 gene associated with human low or failed fertilization. *Molecular Genetics & Genomic Medicine, 8*(10). doi:10.1002/mgg3.1470

Zhang, K., Wang, Y., Cao, J. F., Xu, X. F., & Hao, G. M. (2022). One case of pregnancy in a spouse of a patient with PLCZ1 gene mutation after oocyte assisted activation and literature review. *Reproductive Medicine Journal*(008), 031.

Zhao, S., Cui, Y., Guo, S., Liu, B., Bian, Y., Zhao, S., Chen, Z., & Zhao, H. (2023). Novel variants in ACTL7A and PLCZ1 are associated with male infertility and total fertilization failure. *Clinical Genetics, 103*(5), 603-608. doi:10.1111/cge.14293
