# Supplementary material for: Clinical Features and PLCZ1 Gene Variants in Two Cases of Male Infertility: A Case Series and Literature Review
Source: Mol Genet Genomic Med. 2026 Jun 15;14(6):e70250. doi: 10.1002/mgg3.70250 (PMC13269656; doi:10.1002/mgg3.70250)
Supplement: Supplementary file 3 — Table S3: Baseline characteristics and treatment outcomes of patients with PLCZ1 mutations. [file MGG3-14-e70250-s001.docx]

**Table S3** Baseline characteristics and treatment outcomes of patients with *PLCZ1* mutations

| Author (year) | Country | IVF/ICSI  cycles | Activator（Time） | Oocytes  retrieved | MII | 0PN | 2PN | ≥3PN | Clinical pregnancy outcome | Normal Fertilization  Rate(%) | Semen volume(ml) | concentration  (10^6^/ml) |
| --- | --- | --- | --- | --- | --- | --- | --- | --- | --- | --- | --- | --- |
| (Heytens et al., 2009) | Belgium | NA | NA | NA | NA | NA | NA | NA | No ET | NA | NA | NA |
| (Escoffier et al., 2016)^*^ | Switzerland | ICSI=1  ICSI=1 | NA | 9  9 | 5  7 | NA  NA | 0  0 | NA  NA | No ET  No ET | 0 | 3 | 150 |
|  |  | ICSI=1 |  | 14 | 8 | NA | 0 | NA | No ET | 0 | 5.8 | 101 |
| (Ferrer-Vaquer et al., 2016) | Spain | NA | NA | NA | NA | NA | NA | NA | No ET | NA | NA | NA |
|  |  | NA |  | NA | NA | NA | NA | NA | No ET | NA | NA | NA |
|  |  | NA |  | NA | NA | NA | NA | NA | No ET | NA | NA | NA |
| (Torra-Massana et al., 2019) | Spain | ICSI  ICSI+AOA | Ionomycin  (10umol/L)  twice,  10 minutes each time,with a 30-minute interval. | 7^a^  12^a^ | 7  12 | 7  NA | 0  9 | 0  NA | No ET  Pregnancy | 0  75.00 | NA | NA |
|  |  | ICSI  ICSI |  | 2  5 | 2  5 | 2  5 | 0  0 | 0  0 | No ET  No ET | 0  0 | NA | NA |
|  |  | ICSI  ICSI+AOA |  | 5^a^  9^a^ | 5  9 | 5  NA | 0  6 | 0  NA | No ET  Pregnancy | 0  66.67 | NA | NA |
|  |  | ICSI  ICSI  ICSI  ICSI  ICSI+AOA |  | 11  2  3  3  11^g^ | 11  2  3  3  11 | 11  2  3  NA  NA | 0  0  0  2  3 | 0  0  0  NA  NA | No ET  No ET  No ET  No ET  Pregnancy | 0  0  0  66.67  27.27 | NA | NA |
|  |  | ICSI  ICSI  ICSI  ICSI  ICSI  ICSI  ICSI+AOA |  | 1  4  3  8  5  7  9^a^ | 1  4  3  8  5  7  9 | 1  4  NA  8  NA  7  NA | 0  0  3  0  3  0  8 | 0  0  NA  0  NA  0  NA | No ET  No ET  No ET  No ET  No ET  No ET  Pregnancy | 0  0  66.67  0  60.00  0  88.89 | NA | NA |
|  |  | ICSI  ICSI  ICSI+AOA |  | 5  3  4 | 5  3  4 | 5  NA  NA | 0  1  3 | 0  NA  NA | No ET  No ET  Pregnancy | 0  33.33  75.00 | NA | NA |
|  |  | ICSI  ICSI+AOA |  | 7^a^  9^a^ | 7  9 | NA  NA | 1  7 | NA  NA | No ET  Pregnancy | 14.29  77.78 | NA | NA |
|  |  | ICSI  ICSI+AOA  ICSI+AOA |  | 4  3  4^a^ | 4  3  4 | 4  3  NA | 0  0  2 | 0  0  NA | No ET  No ET  Pregnancy | 0  0  50.00 | NA | NA |
|  |  | ICSI  ICSI  ICSI  ICSI+AOA  ICSI+AOA |  | 6  12  13  15  6 | 6  12  13  15  6 | 6  NA  13  NA  NA | 0  1  0  6  4 | 0  NA  0  NA  NA | No ET  No ET  No ET  NP  NP | 0  8.33  0  40.00  66.67 | NA | NA |
|  |  | ICSI  ICSI  ICSI+AOA |  | 10  8  16 | 10  8  16 | 10  8  NA | 0  0  14 | 0  0  NA | No ET  No ET  Pregnancy | 0  0  87.50 | NA | NA |
|  |  | ICSI  ICSI  ICSI |  | 3^a^  3^a^  8^a^ | 3  3  8 | NA  NA  NA | 1  1  1 | NA  NA  NA | No ET  No ET  No ET | 33.33  33.33  12.50 | NA | NA |
|  |  | ICSI  ICSI+AOA |  | 7^a^  12^a^ | 7  12 | NA  NA | 1  5 | NA  NA | No ET  NP | 14.29  41.67 | NA | NA |
|  |  | ICSI  ICSI  ICSI  ICSI  ICSI+AOA  ICSI+AOA |  | 2  4  4  10  9^a^  9^a^ | 2  4  4  10  9  9 | 2  4  4  NA  NA  NA | 0  0  0  5  5  6 | 0  0  0  NA  NA  NA | No ET  No ET  No ET  No ET  NP  NP | 0  0  0  50.00  55.56  66.67 | NA | NA |
| (Dai et al., 2019) | China | NA  ICSI+AOA=1 | A23187  (10umol/L) 5 minutes. | 20  20 | 17  18 | NA  NA | 0  16 | NA  NA | No ET  No ET | 0  88.89 | 1.5 | 58 |
|  |  | NA  ICSI+AOA=1 |  | 18  14 | 15  6 | NA  NA | 0  2 | NA  NA | No ET  No ET | 0  33.3 | 1.5 | 65 |
|  |  | NA |  | 14 | 13 | NA | 1 | NA | No ET | 7.69 | 1.7 | 58 |
| (Wang et al., 2020) | China | ICSI=1  ICSI+AOA=1  ICSI+AOA=1 | NA | 13  9  2 | 10  8  2 | 0  0  0 | 0  3  2 | 0  0  0 | No ET  NP  No ET | 0  37.5  100 | NA | NA |
| (Mu et al., 2020) | China | IVF=1  ICSI=1  ICSI=1  ICSI=1  ICSI=1  ICSI+AOA=1 | Ionomycin (10umol/L) 10 min). | 10  7  6  2  1  4 | 10  7  6  2  1  2 | NA  NA  NA  NA  NA  0 | 2  1  1  0  0  2 | NA  NA  NA  NA  NA  0 | No ET  No ET  No ET  No ET  No ET  No ET | 20.00  14.29  16.67  0  0  100 | NA | NA |
|  |  | IVF=1  ICSI=1  ICSI+AOA=1 |  | 19  16  14 | 17  12  8 | NA  NA  0 | 1  0  8 | NA  NA  0 | No ET  No ET  Pregnancy | 5.88  0  100 | NA | NA |
|  |  | ICSI=1  ICSI=1  ICSI=1  ICSI+AOA=1 |  | 5  13  25  19 | 3  8  21  10 | NA  NA  NA  NA | 2  2  6  9 | NA  NA  NA  NA | NA  NA  NA  NA | 66.67  25.00  28.57  90.00 | NA | NA |
|  |  | ICSI=1  ICSI=1  ICSI=1  ICSI=1  ICSI=1  ICSI+AOA=1 |  | 12  6  5  6  1  28 | 12  6  2  2  0  26 | NA  NA  NA  NA  NA  NA | 0  0  0  0  0  8 | NA  NA  NA  NA  NA  NA | No ET  No ET  No ET  No ET  No ET  Pregnancy | 0  0  0  0  0  30.77 | NA | NA |
| (Yuan et al., 2020) | China | IVF  IVF+ICSI | NA | 8  4 | 6  4 | NA  NA | 0  0 | NA  NA | No ET  No ET | 0  0 | 2.6  3.0 | 14.6  65.0 |
|  |  | IVF+ICSI  IVF+ICSI  ICSI |  | 6  7  14 | 6  6  13 | NA  NA  NA | 2  2  6 | NA  NA  NA | NP  NP  Pregnancy | 33.33  33.33  46.15 | 2.3  0.5  1.5 | 177.0  50.0  130.0 |
| (Yan et al., 2020) | China | IVF/ICSI=3  ICSI+AOA | Ionomycin (10umol/L) 10 min). | NA  14 | 29  10 | NA  NA | 3  8 | NA  NA | NP  Pregnancy | 10.3  80.00 | NA | NA |
|  |  | IVF/ICSI=2  ICSI+AOA |  | NA  17 | 15  13 | NA  NA | 0  11 | NA  NA | No ET  Pregnancy | 0  84.6 | NA | NA |
|  |  | IVF/ICSI=2  ICSI+AOA |  | NA  11 | 24  9 | NA  NA | 0  8 | NA  NA | No ET  NP | 0  88.9 | NA | NA |
|  |  | IVF/ICSI=1 |  | NA | 25 | NA | 0 | NA | No ET | 0 | NA | NA |
|  |  | IVF/ICSI=2  ICSI+AOA |  | NA  13 | 24  13 | NA  NA | 5  10 | NA  NA | NP  Pregnancy | 20.8  76.9 | NA | NA |
| (Yuan et al., 2021) | China | ICSI  ICSI | NA | 33  8 | 22  4 | NA  NA | 0  0 | NA  NA | No ET  No ET | 0  0 | 2.4  3 | 171.3  277.3 |
| (Zhang et al., 2022) | China | IVF/RICSI=1  ICSI=1  ICSI+AOA=1  ICSI+AOA=1^c^  ICSI+AOA=1 | A23187（5umol/L）  10min. | 23  19  12  12 | 23  16  11^b^  11 | 0  0  5  0  0 | 0  1  0  6  10 | 23  0  0  0  0 | No ET  NP  No ET  Pregnancy^d^  Pregnancy | 0  6.3  0  100  90.9 | NA | NA |
| (Cardona Barberán et al., 2023) | Belgium | ICSI=2 | NA | NA | NA | NA | NA | NA | No ET | NA | NA | NA |
| (Bekaert et al., 2023) | Belgium | NA | Ionomycin  (10umol/L)  twice,  10 minutes each time, with a 30-minute interval | NA | NA | NA | NA | NA | No ET | NA | NA | NA |
| (Lin et al., 2023) | China | IVF  ICSI+AOA  ICSI+AOA  ICSI-donor  sperm | NA | 10  18  14  8 | 8  13  10  7 | 1  7  10  0 | 0  4  0  7 | 7  1  0  0 | No ET  Pregnancy  No ET  Pregnancy | 0  30.77  0  100 | 2 | 53 |
|  |  | IVF  ICSI+AOA |  | 8  7 | 4  4 | 1  1 | 2  2 | 1  0 | No ET  NP | 50.00  50.00 | 2.2 | 147 |
|  |  | IVF  ICSI+AOA  ICSI+AOA |  | 11  10  7 | 10  NA  6 | 0  NA  4 | 0  NA  2 | 10  NA  0 | No ET  No ET  Pregnancy | 0  NA  33.33 | 1.8 | 94 |
|  |  | IVF  ICSI  ICSI  ICSI+AOA |  | 18  18  14  16 | NA  13  NA  15 | NA  NA  NA  2 | 6  6  NA  7 | NA  NA  NA  2 | No ET  No ET  No ET  No ET | NA  46.15  NA  46.67 | 2 | 158 |
| (Zhao et al., 2023) | China | IVF  ICSI  ICSI+AOA  ICSI+AOA | A23187  (10umol/L) 15 minutes | 4  3  1  4 | 3  3  1  4 | NA  NA  NA  NA | 0  0  1  1 | NA  NA  NA  NA | No ET  No ET  No ET  NP | 0  0  100  25.00 | NA | NA |
|  |  | ICSI  ICSI+AOA  ICSI+AOA  ICSI+AOA |  | 14  5  9  9 | 14  5  5  9 | NA  NA  NA  NA | 0  2  3  4 | NA  NA  NA  NA | No ET  No ET  NP  No ET | 0  40.00  80.00  44.44 | NA | NA |
| (Peng et al., 2022) | China | IVF  ICSI+AOA | NA | NA  NA | 7  10 | 1  0 | 0  10 | 6  0 | No ET  Pregnancy | 0  100.00 | NA | NA |
|  |  | IVF(RICSI)  ICSI+AOA |  | NA  NA | 11  10 | 11  2 | 0  8 | 0  0 | No ET  Pregnancy | 0  80.00 | NA | NA |
| (Li et al., 2023) | China | IVF=1  ICSI+AOA=1  ICSI+AOA=1^e^ |  | 5  18  6^f^ | 3  12  6 | 3  12  NA | 0  0  NA | 0  0  NA | No ET  No ET  No ET | 0  0  NA | NA | NA |
| (Wu et al., 2024) | China | IVF/RICSI=1  ICSI/  ICSI+AOA=1^h^  ICSI+AOA=1 | A23187  (15 minutes) | 18  18  13 | 17  8  9  9 | 0  6  2  0 | 0  1  2  4 | 12^g^  0  0  0 | No ET  No ET  NP  Pregnancy | 0  12.5  22.2  44.4 | NA | 111.7 |
| (Barberan et al., 2024) | Belgium | ICSI=5  ICSI+AOA=1 | Ionomycin  (10 umol/L)  two 10-min exposures | NA  NA | 65  20 | NA  NA | 5  8 | NA  NA | Pregnancy  Pregnancy | 7.7  40 | NA | NA |
|  |  | ICSI=1  ICSI+AOA=8 |  | NA  NA | 10  63 | NA  NA | 0  43 | NA  NA | No ET  Pregnancy | 0  68.25 | NA | NA |
|  |  | ICSI=2  ICSI+AOA=1 |  | NA  NA | 10  6 | NA  NA | 1  5 | NA  NA | No ET  NP | 10.0  83.33 | NA | NA |
|  |  | ICSI=4  ICSI+AOA=1 |  | NA  NA | 81  15 | NA  NA | 2  12 | NA  NA | NP  Pregnancy | 2.47  80 | NA | NA |
|  |  | ICSI=3  ICSI+AOA=2 |  | NA  NA | 18  13 | NA  NA | 0  4 | NA  NA | No ET  No ET | 0  30.77 | NA | NA |
|  |  | ICSI=3  ICSI+AOA=3 |  | NA  NA | 16  7 | NA  NA | 5  2 | NA  NA | NP  Pregnancy | 31.25  2.57 | NA | NA |
|  |  | ICSI=5  ICSI+AOA=1 |  | NA  NA | 62  13 | NA  NA | 16  8 | NA  NA | Pregnancy  Pregnancy | 25.81  61.54 | NA | NA |
|  |  | ICSI=2  ICSI+AOA=1 |  | NA  NA | 16  13 | NA  NA | 3  8 | NA  NA | NP  Pregnancy | 18.75  61.54 | NA | NA |
|  |  | ICSI=1  ICSI+AOA=1 |  | NA  NA | 8  17 | NA  NA | 0  13 | NA  NA | No ET  Pregnancy | 0  76.47 | NA | NA |
|  |  | ICSI=4  ICSI+AOA=2 |  | NA  NA | 26  16 | NA  NA | 3  10 | NA  NA | Pregnancy  Pregnancy | 11.54  62.50 | NA | NA |
|  |  | ICSI=1  ICSI+AOA=1 |  | NA  NA | 11  18 | NA  NA | 0  14 | NA  NA | No ET  Pregnancy^d^ | 0  77.78 | NA | NA |
|  |  | ICSI=1  ICSI+AOA=5 |  | NA  NA | 4  25 | NA  NA | 1  13 | NA  NA | No ET  Pregnancy | 25  52 | NA | NA |
|  |  | ICSI=2  ICSI+AOA=1 |  | NA  NA | 25  11 | NA  NA | 0  11 | NA  NA | No ET  Pregnancy | 0  100 | NA | NA |
|  |  | ICSI=2  ICSI+AOA=2 |  | NA  NA | 15  31 | NA  NA | 1  14 | NA  NA | No ET  Pregnancy | 6.67  45.16 | NA | NA |
|  |  | ICSI=2  ICSI+AOA=1 |  | NA  NA | 21  7 | NA  NA | 4  7 | NA  NA | Pregnancy  NP | 19.05  100 | NA | NA |
|  |  | ICSI=3  ICSI+AOA=1 |  | NA  NA | 35  9 | NA  NA | 7  6 | NA  NA | Pregnancy  Pregnancy | 20 | NA | NA |
| (Che et al., 2024) | China | IVF=1  ICSI+AOA=1 | A23187  (10 µmol/L)  5 minutes | 13  10 | 10  7 | 0  - | 0  5 | 10  - | No ET  Pregnancy | 0 | 4.1 | 49.9 |
|  |  | IVF=1  IVF=1  ICSI=1  ICSI+AOA=1  ICSI+AOA=1 |  | 5  10  5  3  5 | 5  5  5  3  3 | 2  1  5  0  0 | 0  0  0  1  2 | 3  4  0  0  1 | No ET  No ET  No ET  No ET  Pregnancy | 0  0  0  33.33  66.67 | 2.8 | 45.8 |
| (Tong et al., 2024)^#^ | China | ICSI=1  ICSI+AOA=1 | A23187  (10 µmol/L)  10 minutes | 5  13 | 4  12 | NA  NA | 0  11 | 2  NA | No ET  Pregnancy | 0  91.67 | 3.7 | 68 |
|  |  | IVF=1  ICSI=1  ICSI+AOA=1 |  | 13  10  10 | 13  9  9 | NA  9  NA | 0  0  7 | 12  0  NA | No ET  No ET  Pregnancy | 0  0  77.78 | 3.6 | 49 |
|  |  | ICSI=1  ICSI+AOA=1 |  | 20  10 | 19  6 | 0  NA | 0  4 | 19  NA | No ET  Pregnancy | 0  66.67 | 4 | 59 |
| (Li et al., 2024) | China | ICSI=1 | NA | NA | 17 | NA | 0 | NA | No ET | 0 | 2.7 | 20.1 |
|  |  | ICSI=1 |  | NA | 44 | NA | 4 | NA | NP | 9.09 | 1.7 | 90.1 |
|  |  | ICSI=1  ICSI+AOA=1 |  | NA | 12  12 | NA  NA | 0  8 | NA  NA | No ET  Pregnancy | 0  66.67 | 2.5 | 40.5 |
|  |  | ICSI=1  ICSI+AOA=1  ICSI+AOA=1 |  | NA | 2  2  7 | NA  NA  NA | 0  2  6 | NA  NA  NA | No ET  No ET  Pregnancy | 0  100  85.71 | 3.0 | 235 |
|  |  | IVF=1  ICSI=1  ICSI+AOA=1 |  | NA | 11  9  9 | NA  NA  NA | 4  0  2 | NA  NA  NA | No ET  No ET  No ET | 36.36  0  22.22 | 4.3 | 96 |
| (Hu et al., 2025) | China | IVF=1  IVF=1  ICSI=1  ICSI+AOA=1 | Ionomycin  (10 umol/L)  10 minutes | 7  11  11  9 | 5  10  11  9 | 0  0  11  1 | 2?  0  0  7 | 2  10  0  0 | No ET  No ET  No ET  Pregnancy^d^ | 80  0  0  77.8 | 4.6 | 49 |
| (Our study 2025) | China | ICSI=1  ICSI+AOA=1 | A23187  (10 µmol/L)  15 minutes | 29  25 | 27  22 | 27  6 | 0  16 | 0  6 | No ET  Pregnancy | 0  72.73 | 3.1 | 16.45 |
|  |  | ICSI=1  ICSI+AOA=1 | Ionomycin  (10 umol/L)  15 minutes | 5  8 | 3  5 | 1  1 | 0  4 | 2  0 | No ET  Pregnancy | 0  80.00 | 2.3 | 87.44 |

Notes: a: Donor; b: Freezing of 6 eggs; c: Oocyte thawing cycle;; d: Biochemical pregnancy; e: Ovulation cycle; f: Received 6 eggs; g: In the same cycle, after low fertilization with IVF, RICSI was performed. Among them, 5 cases used multiple PN sources for IVF, and 7 cases used multiple PN sources for RICSI; h: During the same period, 8 embryos underwent ICSI and 9 embryos underwent ICSI combined with AOA; ET: embryo transfer; PR: Progressive motility rate; 2PN: 2 pronuclei; MII: metaphase II oocytes; AOA: artificial oocyte activation; NA: not available; NP: No pregnancy.

**REFERENCES**

Barberan, A. C., Guggilla, R. R., Colenbier, C., Van der Velden, E., Rybouchkin, A., Stoop, D., Leybaert, L., Coucke, P., Symoens, S., Boel, A., Meerschaut, F. V., & Heindryckx, B. (2024). High rate of detected variants in male PLCZ1 and ACTL7A genes causing failed fertilization after ICSI. *Human Reproduction Open, 2024*(4). doi:10.1093/hropen/hoae057

Bekaert, B., Boel, A., De Witte, L., Vandenberghe, W., Popovic, M., Stamatiadis, P., Cosemans, G., Tordeurs, L., De Loore, A.-M., Chuva de Sousa Lopes, S. M., De Sutter, P., Stoop, D., Coucke, P., Menten, B., & Heindryckx, B. (2023). Retained chromosomal integrity following CRISPR-Cas9-based mutational correction in human embryos. *Molecular Therapy, 31*(8), 2326-2341. doi:10.1016/j.ymthe.2023.06.013

Cardona Barberán, A., Bonte, D., Boel, A., Thys, V., Paredis, R., Machtelinckx, F., De Sutter, P., De Croo, I., Leybaert, L., Stoop, D., Coucke, P., Vanden Meerschaut, F., & Heindryckx, B. (2023). Assisted oocyte activation does not overcome recurrent embryo developmental problems. *Human Reproduction, 38*(5), 872-885. doi:10.1093/humrep/dead051

Che, J., Wu, H., Zeng, S., Wu, Y., Dai, J., Cheng, D., Gong, F., Lu, G., Lin, G., & Dai, C. (2024). Defects in phospholipase C zeta cause polyspermy and low fertilization after conventional IVF:not just ICSI failure. *The Asian Journal of Menology (English Edition), 26*(2), 175-182.

Dai, J., Dai, C., Guo, J., Zheng, W., Zhang, T., Li, Y., Lu, C., Gong, F., Lu, G., & Lin, G. (2019). Novel homozygous variations in PLCZ1 lead to poor or failed fertilization characterized by abnormal localization patterns of PLCζ in sperm. *Clinical Genetics, 97*(2), 347-351. doi:10.1111/cge.13636

Escoffier, J., Lee, H. C., Yassine, S., Zouari, R., Martinez, G., Karaouzène, T., Coutton, C., Kherraf, Z.-e., Halouani, L., Triki, C., Nef, S., Thierry-Mieg, N., Savinov, S. N., Fissore, R., Ray, P. F., & Arnoult, C. (2016). Homozygous mutation of PLCZ1 leads to defective human oocyte activation and infertility that is not rescued by the WW-binding protein PAWP. *Human Molecular Genetics, 25*(5), 878-891. doi:10.1093/hmg/ddv617

Ferrer-Vaquer, A., Barragan, M., Freour, T., Vernaeve, V., & Vassena, R. (2016). PLCζ sequence, protein levels, and distribution in human sperm do not correlate with semen characteristics and fertilization rates after ICSI. *Journal of Assisted Reproduction & Genetics, 33*(6), 747-756.

Heytens, E., Parrington, J., Coward, K., Young, C., Lambrecht, S., Yoon, S. Y., Fissore, R. A., Hamer, R., Deane, C. M., Ruas, M., Grasa, P., Soleimani, R., Cuvelier, C. A., Gerris, J., Dhont, M., Deforce, D., Leybaert, L., & De Sutter, P. (2009). Reduced amounts and abnormal forms of phospholipase C zeta (PLC) in spermatozoa from infertile men. *Human Reproduction, 24*(10), 2417-2428. doi:10.1093/humrep/dep207

Hu, Y. Y., Wang, Q. Y., Yan, X., Sun, Z. F., Zhang, X., Zhang, Y., & Zhang, C. j. (2025). One case of PLCZ1 compound heterozygous mutation patient underwent ICSI combined with oocyte activation assistance, and a literature review was conducted. *Chinese Journal of Reproduction and Contraception, 45*(6), 618-621. doi:10.3760/cma.j.cn101441-20241105-00405

Li, C. H., Cheng, D. K., Ren, H. Q., & Li, C. Y. (2023). A Case Report of Complete Fertilization Failure Caused by Molecular Genetic Factors. *Journal of Maternal and Child Health, 2*(7), 72-74.

Li, Q., Guo, J. C., Huang, G. L., Wu, N., Chen, S., Dai, J., Zhang, X. G., Zhang, G. H., Zhi, W. W., Yan, J. R., Zheng, R., Yan, F., Yan, Z., Wu, L., Wu, S. X., Ji, Z. L., Zeng, J. Z., Lin, G., Li, B., & Xu, W. M. (2024). Novel PLCZ1 compound heterozygous mutations indicate gene dosage effect involved in total fertilisation failure after ICSI. *Reproduction, 168*(4). doi:10.1530/rep-23-0466

Lin, Y., Huang, Y., Li, B., Zhang, T., Niu, Y., Hu, S., Ding, Y., Yao, G., Wei, Z., Yao, N., Yao, Y., Lu, Y., He, Y., Zhu, Q., Zhang, L., & Sun, Y. (2023). Novel mutations in PLCZ1 lead to early embryonic arrest as a male factor. *Frontiers in Cell and Developmental Biology, 11*. doi:10.3389/fcell.2023.1193248

Mu, J., Zhang, Z., Wu, L., Fu, J., Chen, B., Yan, Z., Li, B., Zhou, Z., Wang, W., Zhao, L., Dong, J., Kuang, Y., Sun, X., He, L., Wang, L., & Sang, Q. (2020). The identification of novel mutations in PLCZ1 responsible for human fertilization failure and a therapeutic intervention by artificial oocyte activation. *Molecular Human Reproduction, 26*(2), 80-87. doi:10.1093/molehr/gaaa003

Peng, Y., Lin, Y., Deng, K., Shen, J., Cui, Y., Liu, J., Yang, X., & Diao, F. (2022). Mutations in PLCZ1 induce male infertility associated with polyspermy and fertilization failure. *Journal of Assisted Reproduction and Genetics, 40*(1), 53-64. doi:10.1007/s10815-022-02670-2

Tong, K., Liu, W., Sun, L., Liu, D., Xiang, Y., Li, C., Chai, L., Chen, K., Huang, G., & Li, J. (2024). Novel PLCZ1 mutation caused polyspermy during in vitro fertilization. *Asian Journal of Andrology, 26*(4), 389-395. doi:10.4103/aja202376

Torra-Massana, M., Cornet-Bartolomé, D., Barragán, M., Durban, M., Ferrer-Vaquer, A., Zambelli, F., Rodriguez, A., Oliva, R., & Vassena, R. (2019). Novel phospholipase C zeta 1 mutations associated with fertilization failures after ICSI. *Human Reproduction, 34*(8), 1494-1504. doi:10.1093/humrep/dez094

Wang, F., Zhang, J., Kong, S., Li, C., Zhang, Z., He, X., Wu, H., Tang, D., Zha, X., Tan, Q., Duan, Z., Cao, Y., & Zhu, F. (2020). A homozygous nonsense mutation of PLCZ1 cause male infertility with oocyte activation deficiency. *Journal of Assisted Reproduction and Genetics, 37*(4), 821-828. doi:10.1007/s10815-020-01719-4

Wu, T. H., Chen, C. M., Sun, Q., Xu, F., & Mo, M. L. (2024). One case of PLCZ1 compound heterozygous mutation leading to low IVF multiple-nucleus fertilization and ICSI fertilization. *Chinese Journal of Eugenics and Genetics, 32*(5), 1034-1039.

Yan, Z., Fan, Y., Wang, F., Yan, Z., Li, M., Ouyang, J., Wu, L., Yin, M., Zhao, J., Kuang, Y., Li, B., & Lyu, Q. (2020). Novel mutations in PLCZ1 cause male infertility due to fertilization failure or poor fertilization. *Human Reproduction, 35*(2), 472-481. doi:10.1093/humrep/dez282

Yuan, P., Yang, C., Ren, Y., Yan, J., Nie, Y., Yan, L., & Qiao, J. (2021). A novel homozygous mutation of phospholipase C zeta leading to defective human oocyte activation and fertilization failure. *Human Reproduction, 35*(4), 977-985. doi:10.1093/HUMREP/DEZ293

Yuan, P., Zheng, L. Y., Liang, H., Lin, Q. Y., Ou, S. B., Zhu, Y. Q., Lai, L. H., Zhang, Q. X., He, Z. Y., & Wang, W. J. (2020). Novel mutations in the PLCZ1 gene associated with human low or failed fertilization. *Molecular Genetics & Genomic Medicine, 8*(10). doi:10.1002/mgg3.1470

Zhang, K., Wang, Y., Cao, J. F., Xu, X. F., & Hao, G. M. (2022). One case of pregnancy in a spouse of a patient with PLCZ1 gene mutation after oocyte assisted activation and literature review. *Reproductive Medicine Journal*(008), 031.

Zhao, S., Cui, Y., Guo, S., Liu, B., Bian, Y., Zhao, S., Chen, Z., & Zhao, H. (2023). Novel variants in ACTL7A and PLCZ1 are associated with male infertility and total fertilization failure. *Clinical Genetics, 103*(5), 603-608. doi:10.1111/cge.14293
